# The Dual-Purpose Hen as a Chance: Avoiding Injurious Pecking in Modern Laying Hen Husbandry

**DOI:** 10.3390/ani10010016

**Published:** 2019-12-19

**Authors:** Mona Franziska Giersberg, Birgit Spindler, Bas Rodenburg, Nicole Kemper

**Affiliations:** 1 Adaptation Physiology Group, Wageningen University & Research, 6700 AH Wageningen, The Netherlands; 2 Institute for Animal Hygiene, Animal Welfare and Farm Animal Behaviour, University of Veterinary Medicine Hannover, 30173 Hannover, Germany; birgit.spindler@tiho-hannover.de (B.S.); nicole.kemper@tiho-hannover.de (N.K.); 3 Department of Animals in Science and Society, Faculty of Veterinary Medicine, Utrecht University, 3508 TD Utrecht, The Netherlands; t.b.rodenburg@uu.nl

**Keywords:** animal welfare, laying hens, plumage loss, integument damage, injury, feather pecking, cannibalism

## Abstract

**Simple Summary:**

Dual purpose chickens are one solution to the killing of male day-old chickens from the layer strains. However, modern laying hen husbandry faces further challenges, for instance, the frequent occurrence of injurious pecking. This behavior is seen as a sign of stress in the offending birds, it causes pain and damage in the victims, and thus impairs the health and welfare of the whole flock. In this study, the behavior of conventional laying hens and dual-purpose hens was evaluated comparatively by assessing the status of their feathers and skin over time. All hens were housed and managed under semi-commercial conditions. Severe feather loss and skin lesions due to injurious pecking only occurred in the conventional layer flocks. Therefore, keeping dual-purpose hens may also be an alternative approach to overcome damaging behaviors in laying hen husbandry.

**Abstract:**

Dual-purpose strains, with hens housed for egg laying and roosters kept for meat production are one alternative to the killing of male day-old chickens. However, dual-purpose hens seem to have additional advantages compared to conventional layers, for instance, a lower tendency to develop behavioral disorders, such as feather pecking and cannibalism. In the present study, three batches of about 1850 conventional layers (Lohmann Brown plus, LB+) and 1850 dual-purpose hens (Lohmann Dual, LD) each, all of them with untrimmed beaks, were observed during production (20–71 (56) weeks of life) in a semi-commercial aviary system. The aim was to investigate whether the hybrid and batch affected the occurrence of injurious pecking, and to identify a detailed time course of the damage caused by this behavior. Therefore, the hens’ plumage and skin condition were assessed as an indicator by means of a visual scoring method. The LB+ hens had higher production performances and higher mortality rates compared to the LD hens. Plumage loss in the LB+ flocks started at 23 to 25 weeks of age, and deteriorated continuously. The LD hens showed only moderate feather loss on the head/neck region, which started at 34 to 41 weeks and remained almost constant until the end of the observations. Compared to feather loss, injuries occurred in the LB+ hens with a delay of several weeks, with a maximum of 8% to 12% of hens affected. In contrast, skin injuries were observed only sporadically in single LD hens. In all batches, hybrid had an effect on the occurrence of feather loss (*p* < 0.05). Within the LB+ strain, the proportions of hens affected by plumage loss and injuries differed among batches (*p* < 0.05), whereas this was not the case in the LD flocks. Thus, severe feather pecking and cannibalism seemed to occur in the conventional layer hybrids but not in the dual-purpose hens, though both genetic strains were raised and managed under the same semi-commercial conditions. Therefore, keeping dual-purpose hens should also be considered as an alternative approach to avoid injurious pecking in laying hen husbandry.

## 1. Introduction

The term “injurious pecking” summarizes several undesirable behaviors of partly different etiology in laying hen husbandry. All of these behaviors have in common that they are directed at conspecifics (i.e., self-mutilation is not included) in which they cause physical damage to varying extents. Feather pecking refers to the non-aggressive pecking at, plucking of, and often also removing and ingesting of the feathers of the recipient [1], and is mainly targeted at the hen’s back, tail, and vent area [2]. Different forms of feather pecking have been defined: Severe feather pecking consists of forceful pecks, often with feathers being pulled out and the victim moving away, whereas gentle feather pecking is described as nibbling at feather tips, and usually does not result in a reaction from the recipient bird [1,3]. In some publications, gentle feather pecking is further subdivided into exploratory gentle feather pecks and stereotyped gentle feather pecking bouts [4,5]. Although gentle feather pecking itself does usually not result in feather loss [3], it may develop into severe feather pecking. Furthermore, tissue pecking frequently emerges from severe feather pecking once denuded areas at the hens’ bodies are further pecked at [1]. Tissue pecking may affect the skin only, or also deeper tissues, which is then also referred to as cannibalism [1]. Other forms of cannibalism, such as vent or toe pecking, often occur irrespectively of feather pecking in hens with intact plumage cover [1,6,7]. In contrast, aggressive pecking, which is mainly directed at the birds’ head/neck region, is not regarded as disorder but rather as normal behavior to establish and maintain dominance hierarchies in groups of hens [1,2]. On commercial farms, the plumage condition of hens affected by injurious pecking was found to continuously deteriorate with age [8]. However, little is known about the detailed time course of feather loss and its timely relation to the occurrence of skin lesions during the entire production periods of several laying hen flocks kept at the same farms.

The main welfare issue of injurious pecking is obvious: The occurrence of this behavior in a laying hen flock is indicative of poor or stressful housing and management conditions [2]. Furthermore, depending on the severity and permanence, the recipient birds experience acute and lasting pain [9]. In addition, hens with denuded areas on their bodies may be more susceptible to abrasion, and their thermoregulatory capacity may be impaired. The latter is compensated by an increased feed intake [10,11], leading to economic losses, which may be further exacerbated by high mortality rates due to cannibalism [12,13]. In the past, these consequences of injurious pecking were mainly managed by trimming the chickens’ beaks [14]. However, since this measure itself causes acute and chronic pain in the animals [15,16], many European countries have abolished beak trimming or have planned to end this practice in the near future [17]. Together with the ban on conventional cages in the European Union [18] and a shift towards loose housing systems, this scenario poses several risks for laying hen welfare. It may be more difficult and laborious to control for injurious pecking behavior in loose housing systems, where, due to a larger group size, many potential victims are exposed to one offending hen [19], or where abnormal behaviors can be spread by social learning [20]. On the other hand, these systems also offer the potential to tackle some of the underlying causes of abnormal behaviors, for instance, by providing resources, such as foraging and dustbathing substrates, space, or perches at different heights, whose absence has been associated with the occurrence of injurious pecking [21,22,23,24]. In addition, a well-structured housing system with distinct functional areas offers a more controllable setting, which may enable the hens to better cope with minor environmental or social stressors [2]. Thus, most of the recommended and applied measures to prevent or alleviate injurious pecking in laying hens are based on optimizing the husbandry and management conditions [17].

However, the propensity to develop injurious pecking is not only influenced by resource-related factors but also by genetic background [19]. In observational on-farm studies, the prevalence of feather damage varied among different commercial layer strains [25,26]. In addition, a divergent phenotypic selection on feather pecking behavior was achieved [27]. With methods of molecular genetics, it was also possible to identify quantitative trait loci for injurious pecking behavior [28]. However, since these investigations were carried out either in experimental lines [27,28] or commercial high-yielding layer hybrids, little is known about the incidence of feather pecking and cannibalism in purebred hens [29] or alternative hybrids, such as modern dual-purpose hens, for instance, Lohmann Dual.

The original idea behind keeping these dual-purpose strains nowadays is to avoid the killing of day-old male layer chickens, which raises socioethical and, in some European countries, also legal concerns [30]. Dual-purpose hens should lay a sufficient number of eggs while roosters should show an acceptable fattening performance. Thus, in contrast to raising the male offspring from high-yielding layer hybrids, the aim is that both sexes gain not only intrinsic but also economic value [30]. Due to a negative correlation between reproductive and fattening traits, dual-purpose chickens cannot achieve the production performances of specialized hybrids. Nevertheless, according to a public survey, the dual-purpose concept seems to be one of the favored alternatives to killing day-old chickens [31]. Moreover, anecdotal observations from producers suggest that dual-purpose hens may be less prone to developing injurious pecking behavior. However, these flocks were kept under organic farming conditions and lacked a direct comparison to conventional layer strains.

In a previous study, Giersberg et al. [32] found evidence that dual-purpose hens seem to have a lower tendency to show injurious pecking under commercial housing conditions. However, the main objectives of this investigation were to develop and validate a mere visual method (visual scoring, VSc) for scoring the hens’ plumage and integument condition at a flock level as an alternative to a scientifically established method, which involved the potentially stressful catching and handling of individuals. It was shown that the non-intrusive VSc was reliable for detecting feather loss and skin lesions both in highly (conventional layer hybrids) and slightly (dual-purpose hens) affected flocks. Since the focus was put on the validation of the new assessment method, only one laying period of two groups per genetic strain was studied. However, due to the multiple causes of injurious pecking, the situation in following batches of the same hybrid strain may be different.

The aim of this longitudinal follow-up study was to investigate whether the hybrid strain consistently affected the occurrence of injurious pecking in an on-farm setting, and to identify a detailed time course of the feather and skin damage caused by this behavior. Based on previous research and observations in practice, it was hypothesized that both feather loss and integument lesions indicating injurious pecking behavior would occur to a larger extent in conventional layer hybrids compared to dual-purpose hens. In addition, it was expected that within the hybrid strain, plumage and integument condition might differ among batches.

## 2. Materials and Methods

### 2.1. Animals and Management

The present study was carried out at the research farm “Ruthe” of the University of Veterinary Medicine, Hannover, Germany, and involved a total of 5517 Lohmann Brown plus (LB+, conventional layer hybrids) and 5501 Lohmann Dual hens (LD, dual-purpose hybrids), all of them with untrimmed beaks. LB+ hens had brown feathering and laid brown eggs, whereas LD hens had white feathering and laid cream-colored eggs. The hens were kept in three consecutive batches, and were observed from November 2015 to September 2018.

From one day old to 19 weeks, the LB+ and LD chickens from the first batch were housed in two separate pens, with about 1850 hens each, in the same building at a commercial rearing farm in northern Germany. In the second and the third batch, both hybrids were kept for the first 18 weeks of life in two adjacent pens, again with about 1850 hens each, in the rearing house at the research farm “Ruthe”. At both locations, the pullets were raised according to standard management procedures, which were developed to meet the requirements of the LB+ strain [33]. They had access to feed and water ad libitum, to perches at different heights, wood shavings on the floor, and alfalfa or straw bales. Irrespective of the batch and rearing facility, the housing and management conditions were always kept the same for the two hybrid strains.

At 18 or 19 weeks of life, respectively, the hens were moved to the layer house at “Ruthe”, where, again, both hybrids were subjected to the same housing conditions and standard management procedures, which were established for the LB+ hens [33]. The hens were kept in two compartments per hybrid strain and batch (about 925 hens per compartment, 9 hens/m^2^) [34], which were switched among strains and batches. Each compartment was equipped with an asymmetric aviary system (Natura Nova 270, Big Dutchman, Vechta, Germany). The light regime started with 10L:14D at 18/19 weeks of life and was gradually extended until 14L:10D (week 25). As standard enrichment material, all of the animals were provided with alfalfa bales suspended in hay nets (about one bale every 200 hens). At the first signs of feather pecking or cannibalism, additional measures were taken according to a gradual emergency scheme in all compartments (for details, see [32]) following the recommendations of the working group for laying hens at the Lower Saxony Ministry for Nutrition, Agriculture, and Consumer Protection [35]. Production parameters, such as mortality, laying performance, and feed consumption, were recorded continuously by the farm staff.

### 2.2. Plumage and Integument Scoring

As an indicator for injurious pecking behavior, the plumage and integument condition of the hens was assessed by a mere visual scoring method (VSc), which was developed and validated earlier (for details, see [32]). VSc was performed on a weekly basis, resulting in 51 observation days in batch 1 (20–71 weeks of life), 47 observation days in batch 2 (20–69 weeks of life), and 25 observation days in batch 3 (20–56 weeks of life). Missing observation weeks were due to administrative reasons. Owing to the termination of the research project, batch 3 could only be studied until 56 weeks of life. The hens (n = 200/hybrid and study day) were scored on five distinct body parts (head/neck, back, tail, wing, breast/belly) for feather loss and injuries using a five- and four-point scale, respectively (Table 1, [32]). Plumage loss was evaluated according to its degree; the type of feathers lost was not distinguished (flight feathers, other contour feathers, semiplumes). The definition of injuries focused on fresh or crusted lesions affecting the skin only or also deeper tissues, whereas scars were not considered. Further visible abnormalities on the hens’ bodies, particularly vent and toe lesions, were recorded without a fixed scoring scheme. All of the hens were scored by the same observer, and compartments were visited in a different order on each study day, which was randomly chosen.

### 2.3. Data Presentation and Statistical Analyses

All of the statistical analyses were performed using the software SPSS Statistics (version 25, IBM, Armonk, NY, USA). Owing to the large overall data set, feather scores were summarized according to the following scheme: Intact plumage (former score 0), moderate plumage loss (former scores 1 and 2), and severe plumage loss (former scores 3 and 4). Since feather loss on the breast/belly region could not be identified reliably by the VSc method [32], scores for this body region were excluded from the present analysis. Due to the low incidence of injuries, we only distinguished between intact (former score 0) and damaged integument (former scores 1, 2, and 3). The proportions of hens assigned to the abovementioned categories for each batch, week of life, hybrid, and body region are shown in the Supplementary Materials (Tables S1–S12). For further analysis and data presentation, the following target variables were calculated: Proportion of hens with intact plumage on all body regions, hens with moderate plumage loss on at least one body region, hens with severe plumage loss on at least one body region, and hens with injuries on at least one body region. Subsequently, detailed timelines were plotted for these target variables. Furthermore, inferential statistics were calculated to test for the effects of hybrid and batch on plumage loss and injuries in the studied flocks. Initially, data were examined visually for normality by creating histograms, including the Gaussian distribution curve. The Levene procedure was used to test for homoscedasticity. To obtain normality, data were log transformed. Generalized linear mixed models were calculated to test the effects of hybrid and batch on the target variables. Data were structured by batch x hybrid x compartment (subject) and week as repeated measures. The models contained the fixed effects of hybrid and batch and their interaction. The compartment within hybrid was added as a random effect. Since severe plumage loss and injuries were hardly ever observed in the LD hens, models for these targets included the fixed effect of batch and the random effect of compartment. All post hoc pairwise comparisons were adjusted according to Bonferroni correction. Differences between the tested parameters were considered to be significant if *p*-values were <0.05.

### 2.4. Ethical Note

The present study complies with the requirements of the ethical guidelines of the International Society of Applied Ethology [36]. All of the animals were housed according to EU [18] and national law [37,38]. In compliance with European Directive 2010/63/EU Article 1 5.v(f) [39], the experiments did not imply any invasive treatment of the hens.

## 3. Results

### 3.1. Production Records Throughout Lay

Basic production data for the end of the respective study period of the three batches of LB+ and LD hens are provided in Table 2. During the entire laying periods, the health status of all flocks was good. Neither antimicrobial nor other veterinary treatment was necessary.

### 3.2. Time Course of Plumage Loss and Injuries in LB+ and LD Hens

**Figure 1:**
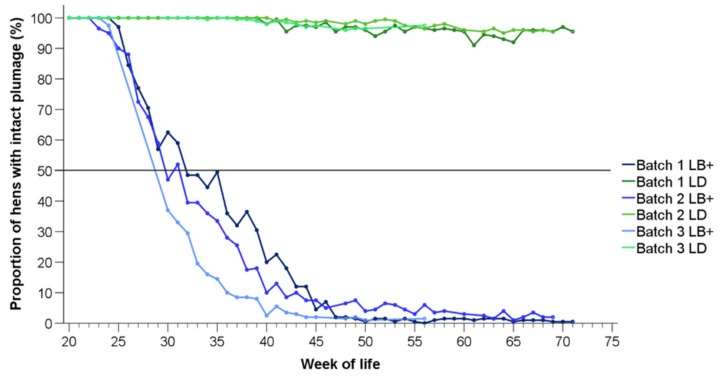
Proportions of LB+ (blue lines) and LD hens (green lines) from three consecutive batches (batch 1–3) with intact plumage on the whole body (*n* = 200 hens/hybrid strain and week).

In this section, the summarized target variables (i.e., proportion of hens with intact plumage on all body regions, hens with moderate plumage loss on at least one body region, hens with severe plumage loss on at least one body region, and hens with injuries on at least one body region) are plotted as time lines per batch and hybrid. The detailed proportions of LB+ and LD hens with different plumage status on the four distinct body regions are provided in the Supplementary Materials (Tables S1–S12). Figure 1 shows the proportions of LB+ and LD hens with completely intact feather cover depending on age. In both hybrid strains and in all batches, the feather cover of the hens was totally intact at the beginning of the observation period (20 weeks of life). In the LB+ flocks, plumage loss started in week 23 (batch 2), week 24 (batch 3), and, at the latest, in week 25 (batch 1). In the same weeks, the plumage of the LD hens in all of the batches was still intact. The plumage condition in the LB+ hens deteriorated with age. Therefore, depending on the batch, 50% of the individuals in the LB+ flocks were affected by feather loss at 30 to 32 weeks of life at the latest. At the end of the observation period, there were hardly any LB+ hens (less than 2% of the flock) with completely intact plumage in all of the examined batches. In contrast, in the LD hens, feather loss was observed for the first time in week 34 (batch 3) to week 41 (batch 2). The proportions of LD hens with intact plumage remained relatively constant thereafter, and did not decrease further than 92% (week 65, batch 1).

**Figure 2:**
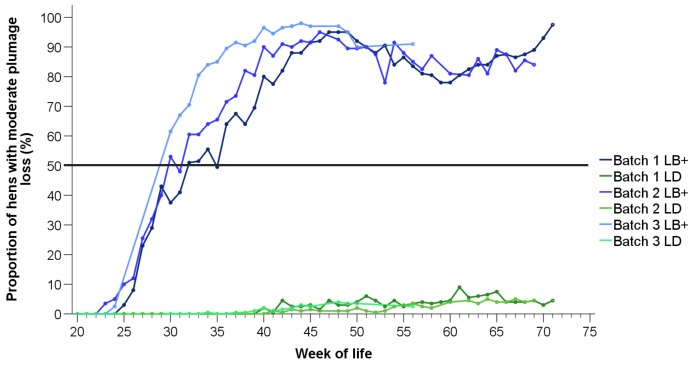
Proportions of LB+ (blue lines) and LD hens (green lines) from three consecutive batches (batch 1–3) with moderate plumage loss on at least one body region (*n* = 200 hens/hybrid strain and week).

The time course of moderate feather loss on at least one body region is presented in Figure 2. For the LB+ flocks, the lines represent a reversal of those in Figure 1, until feather loss on the respective body region turned into “severe”, or the same hen was affected by moderate plumage loss on one body region and by severe feather loss on another (starting at 37–45 weeks). In the LD hens, the proportions of hens with moderate plumage loss (Figure 2) reflect a precise reversal of the proportions of hens with intact feather cover (Figure 1), since plumage loss in this hybrid strain occurred exclusively to a moderate extent and on one body part only (head/neck region, Tables S4–S6).

**Figure 3:**
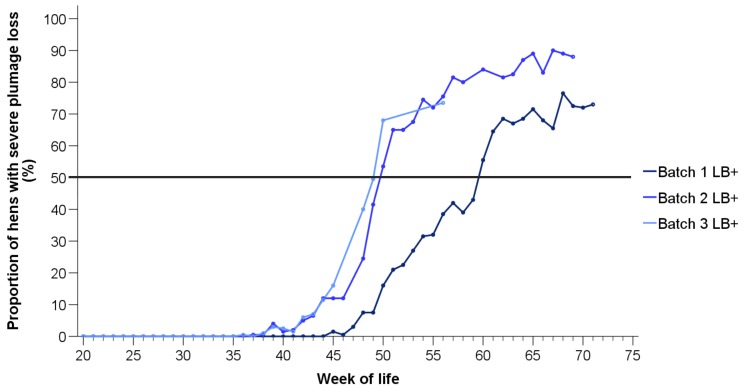
Proportions of LB+ hens from three consecutive batches (batch 1–3) with severe plumage loss on at least one body region (*n* = 200 hens/week).

As stated before, severe plumage loss was not observed in the LD hens. Therefore, the proportions of hens with >50% of the feathers missing on at least one body part are presented for the LB+ flocks only (Figure 3). Severe plumage loss started earlier and affected a larger proportion of hens in the second and the third batch (onset: 37 weeks) compared to the first batch (onset: 45 weeks). Similar to moderate plumage loss, the proportions of animals showing severe feather loss increased with age in all three batches. At 48 to 60 weeks of life, 50% of the LB+ hens were affected by severe plumage loss.

**Figure 4:**
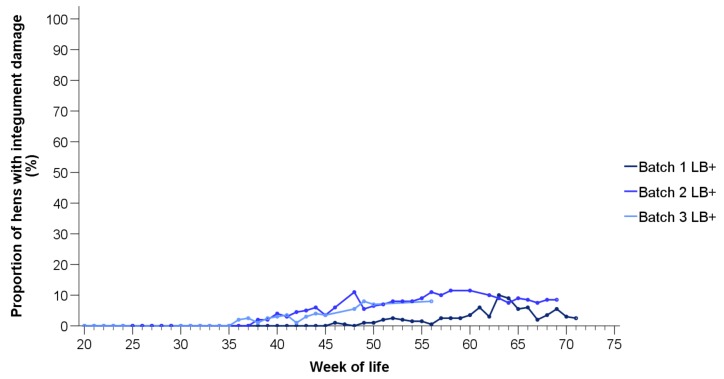
Proportions of LB+ hens from three consecutive batches (batch 1–3) with injuries on at least one body region (*n* = 200 hens/week).

Injuries indicating cloacal or toe cannibalism were not observed in either of the two hybrid strains. In the LD hens, only one hen with skin lesions was found in batch 3 at 43 weeks of life (Table S12). Therefore, time courses of the injuries are shown for the LB+ flocks only (Figure 4). Compared to feather loss, injuries occurred in the LB+ hens with a delay of 12 (batch 3), 15 (batch 2), and 21 weeks (batch 1), respectively. Among the batches, different patterns can be found. In the first batch, for instance, a peak of injured hens was observed (week 63, 10% of the hens affected), whereas the proportion of hens with injuries in the second batch remained at a level of over 5% from the 46th week onwards.

### 3.3. Effects of Hybrid and Batch on Plumage and Integument Condition in LB+ and LD Flocks

As expected from the descriptive time lines, the proportion of hens with completely intact plumage (i.e., a score of 0 on all body parts) was affected by hybrid in the first (t(490) = 24.84, *p* < 0.05), the second (t(490) = 34.11, *p* < 0.05), and the third batch (t(490) = 49.95, *p* < 0.05), respectively. In all of the observed flocks, there were more LD than LB+ hens with fully intact feather cover. Within the hybrid, effects of batch were found in the LB+ strain, with more hens with completely intact plumage in the first compared to the second batch (t(490) = 9.27, *p* < 0.05), in the first compared to the third batch (t(490) = 25.91, *p* < 0.05), and in the second compared to the third batch (t(490) = 16.71, *p* < 0.05). No effect of batch was observed within the LD flocks.

Similar results were obtained for the proportions of hens with moderate plumage loss on at least one body region, which were higher in the LB+ than in the LD flocks in all batches (batch 1: t(490) = 19.28, *p* < 0.05; batch 2: t(490) = 20.92, *p* < 0.05; batch 3: t(490) = 19.19, *p* < 0.05). Pairwise comparisons showed that more LB+ hens were affected in the third compared to the first (t(490) = 9.86, *p* < 0.05) and the second batch (t(490) = 8.01, *p* < 0.05), and in the second compared to the first batch (t(490) = 2.37, *p* < 0.05). In the LD strain, the proportions of hens affected by moderate plumage loss did not differ among batches.

As mentioned earlier, severe plumage loss was not observed in the LD, and in the LB+ hens before 36 weeks of life. Therefore, the influence of batch was only tested in the LB+ flocks at a later age. From the 36th week onwards, more LB+ hens with severe plumage loss were found in batch 2 (t(165) = 20.01, *p* < 0.05) and 3 (t(165) = 15.11, *p* < 0.05) compared to batch 1, whereas no difference was detected between batch 2 and 3 (t(165) = 1.27, *p* = 0.20).

Injuries indicating cloacal or toe cannibalism were not observed in either of the two hybrid strains. Similar to severe plumage loss, injuries in the LB+ flocks occurred from the 36th week of life onwards. Thus, the following results refer to the LB+ strain at 36 weeks or older. The proportions of injured hens differed among all batches, with more animals affected in the second compared to the first (t(165) = 20.73, *p* < 0.05) and the third batch (t(165) = 3.50, *p* < 0.05), and in the third compared to the first batch (t(165) = 3.82, *p* < 0.05).

## 4. Discussions

The present study provides a longitudinal, comparative record of plumage and integument condition of dual-purpose hens (LD) and conventional layer hybrids (LB+) kept under semi-commercial housing and management conditions. As hypothesized, severe feather loss and skin lesions only occurred in the LB+ hens. Within the hybrid strain, batch influenced the prevalence of damage in the LB+ but not in the LD flocks.

Feather loss and injuries, particularly on the bird’s back, rump, and tail, are valid indicators of injurious pecking behavior in laying hens [3]. These can be measured in adequate detail by non-intrusive, visual scoring methods, both at an individual [40] and at the flock level [32]. The main advantage of VSc methods is that they do not require catching and handling, which is potentially stressful for the hens, especially in flocks that show agitation or high levels of fear for humans. Since VSc is less disruptive for the animals and less time consuming for the observer, and it is suitable for regular flock monitoring and longitudinal on-farm research with close inspection intervals. Thus, the depiction of a detailed time course of plumage and integument damage becomes possible. The risk of missing feather loss on body parts that might be challenging to assess visually was only confirmed for the breast/belly region [32]. However, since bald patches on the breast/belly could also not be related to the proportion of pecks that were directed to this body part [3], it was excluded from the present calculations.

In all of the observed LB+ flocks, plumage loss started only a few weeks after transfer to the laying hen house and deteriorated rapidly thereafter, so that at 30 to 32 weeks of life, half of the hens of each batch were affected. This is in line with previous investigations under commercial [8] and experimental conditions [3]. All the hens observed by Bright [8] had intact feather cover upon arrival at the laying farm. However, in 39% of the flocks, plumage loss started rapidly thereafter, and at about 40 weeks, the presence of feather pecking was clearly visible. Similar to the present study, plumage condition in these flocks continued to deteriorate until the end of the production period [8]. No stagnation of damage was observed at any age. In the LB+ flocks, the proportion of hens with intact plumage seemed to improve occasionally between two weeks, for instance, in the first batch between the 29 and the 30 weeks of life, in which 57% and 63% of the birds were scored with no damage. This may be likely explained by variations of the sample on each study day. Since the present assessments were carried out at the flock level, the hens scored per week belonged to the same group but were not necessarily the same individuals [32]. Besides the proportions of affected LB+ hens, the severity of feather loss in individual animals also increased with increasing flock age, which has been reflected indirectly by previous studies [8,41]. However, most on-farm studies did not consider injuries as an indicator for prolonged injurious pecking at denuded body parts of conspecifics [8,25,41,42]. Under experimental conditions, Bilčík and Keeling [3] found the first skin lesions in birds of 22 weeks of age. In contrast, injuries in the LB+ hens started at 36 to 46 weeks. However, the hens in the study by Bilčík and Keeling [3] showed feather loss already at an age of 18 weeks. In the present investigations, skin injuries in the LB+ hens occurred with a delay of 12 to 21 weeks relative to the first plumage loss, and one week before to one week after severe feather loss was detected. This supports the hypothesis that severe feather pecking can turn into tissue pecking, and thus into cannibalism [1]. Within the LB+ strain, an effect of batch was found for the proportion of hens with intact plumage, and for the proportions of hens affected by feather loss of different severity, and injuries. However, interpretation of this effect is difficult, since the present study did not analyze distinct influencing factors for the occurrence of injurious pecking but can rather be regarded as a longitudinal system comparison between two hybrid strains. Previous long-term research on risk factors for feather pecking behavior was carried out during one production period of a number of flocks at different farms, and did not take into account several consecutive flocks at one farm [25,41].

In contrast, none of the above described patterns was found in any of the LD flocks. In the LD hens, slight feather loss was observed on the head/neck, starting from 34 to 41 weeks of age. This may be explained by aggressive pecking behavior, which was found to cause feather loss on the head [3]. Furthermore, a larger proportion of aggressive pecks was actually observed in the LD compared to the LB+ hens, which in turn showed more severe feather pecking behavior, in the functional area of the nest boxes [34]. However, at all assessment days, at least 92% of the LD hens showed intact plumage cover. Although the third batch could only be studied until 56 weeks of age, it is unlikely that the hens developed injurious pecking behavior thereafter [8].

Correspondingly, hybrid effects were found for the proportions of hens with intact plumage and moderate feather loss in all batches. Due to the absence of severe plumage loss and injuries in the LD hens, it was not possible to test these variables for hybrid effects. Previous research confirmed a genetic background of the development and expression of injurious pecking behavior (reviewed in [2,43]). The divergent phenotypic selection for feather pecking behavior over several generations formed the high and low feather pecking lines [27], whose descendants have been used in basic behavioral and physiological research since [5]. However, it is often not clear how these behavioral characteristics are connected to other traits, such as production traits, and to which extent feather pecking traits are therefore present in and expressed by commercially available laying hen hybrids. Results on the link between laying performance and feather pecking, for instance, are inconsistent. In one study, low-yielding purebred New Hampshire hens showed less feather pecking than commercial hybrids [44], whereas in another investigation, hens of the local Danish landrace expressed more injurious pecking compared to high-yielding layer hybrids [27]. A further trait that has been associated with the occurrence of feather pecking in commercial flocks is plumage coloration. Keeling et al. [45] found that hens that are homozygous for a pigmentation causing the wild-type allele were more vulnerable to feather pecking than birds with a dominant white allele. Similarly, Bright [46] observed less damage due to injurious pecking in white compared to grey and black feathered birds from the same layer strain. Since the LB+ hens were brown layers, and the LD hens had white feathers, differences in plumage coloration might have accounted for the observed hybrid effects to a certain degree. Further investigations should disentangle the effects of plumage coloration and bird strain, for instance, by comparing dual-purpose hens with white-feathered conventional layer hybrids. In addition, the LD hens should be characterized for other traits that have been linked to the expression of injurious pecking behavior, such as fearfulness or coping strategy [19].

As stated before, from the present study one cannot conclude which environmental factors might have triggered the observed feather loss and skin lesions, which indicated the development of injurious pecking in the LB+ flocks. The hens were housed in a semi-commercial setting, which can be regarded as representative for many laying hen farms in Europe. Known risk factors for behavioral disorders were largely eliminated, for instance, by providing permanent access to litter and alfalfa bales as additional standard enrichment material [17]. However, since both hybrids were kept under the same rearing, housing, and management conditions, the LD hens were exposed to the same environmental influencing factors.

Thus, the present study confirms the patterns of the preliminary data set used for validating a method for plumage and integument assessment [32]. Besides avoiding the killing of day-old male chickens, keeping dual-purpose hens has an additional major benefit compared to conventional layer hybrids: A lower prevalence of welfare-relevant damage related to injurious pecking behavior. Although the scientific literature on dual-purpose hens is scarce, further positive aspects of these hybrids have been identified in other fields of research. LD hens were, for instance, less susceptible to natural mixed nematode infections [47], which may be advantageous in alternative housing systems, particularly in those with access to covered verandas or pasture. However, it is important to consider the specific characteristics of dual-purpose hens, for instance, their rather compact morphology or their perching and nesting preferences, when housing them in existing systems designed for conventional layer hybrids [34,48].

The major challenge of the dual-purpose concept is to keep the animals in an ecologically and economically sustainable way. As expected, the LD hens showed a lower production performance in terms of laying rate and number of eggs laid per average hen housed. However, compared to the LB+ hens, the LD hens consumed about 20% less of a standard layer diet in the present study. Furthermore, the average feed consumption in kg per kg of produced egg mass was similar in both hybrids. In addition, feeding LD hens an energy- and nutrient-reduced diet containing 10% lignocellulose was associated with a decrease of body fat content and an improvement of laying performance [49]. Therefore, future research should examine to which extent dual-purpose hens could utilize alternative feed sources, such as byproducts or leftovers from the food production. Moreover, animal welfare is also one aspect of the concept of sustainability in livestock farming [50]. Therefore, it is questionable whether layer flocks, which show a high egg laying performance but at the same time physical damage and high mortality rates of often more than 20% [26], fit into a sustainable concept like this.

The dual-purpose concept must certainly not be regarded as the exclusive approach to prevent injurious pecking effectively and in the short term on commercial farms. Although it might not be applicable for a large share of egg producers to house dual-purpose hens at present, this concept can contribute to a diversification of poultry production towards sustainable, welfare-friendly systems. Other perspectives, such as management approaches, should be further developed and re-evaluated in on-farm contexts [43]. However, when tackling the multifactorial problems of feather pecking and cannibalism it is important not to adopt preventive measures that pose welfare risks themselves, like beak trimming or low light intensities in the hen house [17]. Therefore, existing approaches and recommendations to prevent injurious pecking in laying hens should be continuously refined and complemented with suggestions that seem novel or exceptional, such as keeping dual-purpose hens.

## 5. Conclusions

Keeping dual-purpose hens with untrimmed beaks under standard management procedures in semi-commercial loose housing systems is largely unproblematic regarding the occurrence of damaging behaviors. In three consecutive batches, the dual-purpose hens did not show signs of feather pecking or cannibalism throughout the laying period, whereas the conventional layer hybrids showed an increasing prevalence of feather loss and skin injuries from early to mid lay to late lay. These results indicate that dual-purpose hens may experience higher levels of welfare in aviary systems compared to conventional layer hybrids. Thus, keeping dual-purpose hens should be considered as one alternative approach to avoid injurious pecking in modern laying hen husbandry.

## Figures and Tables

**Table 1:** Description of the scoring scheme used for the assessment of plumage and integument condition in conventional layer hybrids and dual-purpose hens [32].

Parameter/Score	Feather Loss	Injuries
0	No feather loss	No injuries
1	≤25% of the feathers of the body part missing	Single injury of <0.5 cm diameter or length
2	>25% and ≤50% of the feathers of the body part missing	Multiple injuries of <0.5 cm or single injuries of >0.5 cm and ≤1.0 cm
3	>50% and ≤75% of the feathers of the body part missing	Single or multiple injuries of >1.0 cm
4	>75% of the feathers of the body part missing	-

**Table 2:** Production data for three batches of conventional layers (LB+) and dual-purpose hens (LD) at the end of the respective study period (week of life).

Batch	Week of Life	Hybrid	Cumulative Mortality (%)	Laying Rate (%)	Number of Eggs/Average Hen Housed	Average Feed Consumption/d (g)	Average Feed Consumption (kg)/kg Egg Mass
1	19–71	LB+	6.91	84.83	338	116.92	2.19
		LD	2.34	73.56	293	97.54	2.25
2	18–69	LB+	12.06	84.92	322	126.12	2.45
		LD	7.86	67.25	255	97.59	2.52
3	18–56	LB+	8.68	89.24	240	117.97	2.29
		LD	5.02	70.45	190	89.00	2.37

## Supplementary Materials

The following are available online at https://www.mdpi.com/2076-2615/10/1/16/s1, Table S1: Proportions of LB+ and LD hens (*n* = 200/hybrid strain) from batch 1 with intact plumage for four body regions, and the whole body, respectively, Table S2: Proportions of LB+ and LD hens (*n* = 200/hybrid strain) from batch 2 with intact plumage for four body regions, and the whole body, respectively, Table S3: Proportions of LB+ and LD hens (*n* = 200/hybrid strain) from batch 3 with intact plumage for four body regions, and the whole body, respectively, Table S4: Proportions of LB+ and LD hens (*n* = 200/hybrid strain) from batch 1 with moderate plumage loss for four body regions. Min. one body region affected: proportion of hens with at least one body region affected by moderate plumage loss, Table S5: Proportions of LB+ and LD hens (*n* = 200/hybrid strain) from batch 2 with moderate plumage loss for four body regions. Min. one body region affected: proportion of hens with at least one body region affected by moderate plumage loss, Table S6: Proportions of LB+ and LD hens (n = 200/hybrid strain) from batch 3 with moderate plumage loss for four body regions. Min. one body region affected: proportion of hens with at least one body region affected by moderate plumage loss, Table S7. Proportions of LB+ and LD hens (*n* = 200/hybrid strain) from batch 1 with severe plumage loss for four body regions. Min. one body region affected: proportion of hens with at least one body region affected by severe plumage loss, Table S8: Proportions of LB+ and LD hens (*n* = 200/hybrid strain) from batch 2 with severe plumage loss for four body regions. Min. one body region affected: proportion of hens with at least one body region affected by severe plumage loss, Table S9: Proportions of LB+ and LD hens (*n* = 200/hybrid strain) from batch 3 with severe plumage loss for four body regions. Min. one body region affected: proportion of hens with at least one body region affected by severe plumage loss, Table S10: Proportions of LB+ and LD hens (*n* = 200/hybrid strain) from batch 1 with injuries for five body regions. Min. one body region affected: proportion of hens with at least one body region affected by injuries, Table S11: Proportions of LB+ and LD hens (*n* = 200/hybrid strain) from batch 2 with injuries for five body regions. Min. one body region affected: proportion of hens with at least one body region affected by injuries. Table S12: Proportions of LB+ and LD hens (*n* = 200/hybrid strain) from batch 3 with injuries for five body regions. Min. one body region affected: proportion of hens with at least one body region affected by injuries.
