# Supplementary material for: The Dual-Purpose Hen as a Chance: Avoiding Injurious Pecking in Modern Laying Hen Husbandry
Source: Animals (Basel). 2019 Dec 19;10(1):16. doi: 10.3390/ani10010016 (PMC7023478; doi:10.3390/ani10010016)
Supplement: Supplementary file 1 [file animals-10-00016-s001.docx]

**Table S1.** Proportions of LB+ and LD hens (*n* = 200/hybrid strain) from batch 1 with intact plumage for four body regions, and the whole body, respectively.

| **Week of Life** | **Hybrid** | **Body Region** | | | | **Whole Body Intact** |
| --- | --- | --- | --- | --- | --- | --- |
|  |  | **Head/neck** | **Back** | **Tail** | **Wing** |  |
| 20 | LB+ | 100.0 | 100.0 | 100.0 | 100.0 | 100.0 |
|  | LD | 100.0 | 100.0 | 100.0 | 100.0 | 100.0 |
| 21 | LB+ | 100.0 | 100.0 | 100.0 | 100.0 | 100.0 |
|  | LD | 100.0 | 100.0 | 100.0 | 100.0 | 100.0 |
| 22 | LB+ | 100.0 | 100.0 | 100.0 | 100.0 | 100.0 |
|  | LD | 100.0 | 100.0 | 100.0 | 100.0 | 100.0 |
| 23 | LB+ | 100.0 | 100.0 | 100.0 | 100.0 | 100.0 |
|  | LD | 100.0 | 100.0 | 100.0 | 100.0 | 100.0 |
| 24 | LB+ | 100.0 | 100.0 | 100.0 | 100.0 | 100.0 |
|  | LD | 100.0 | 100.0 | 100.0 | 100.0 | 100.0 |
| 25 | LB+ | 100.0 | 97.0 | 100.0 | 100.0 | 97.0 |
|  | LD | 100.0 | 100.0 | 100.0 | 100.0 | 100.0 |
| 26 | LB+ | 100.0 | 84.5 | 100.0 | 100.0 | 84.5 |
|  | LD | 100.0 | 100.0 | 100.0 | 100.0 | 100.0 |
| 27 | LB+ | 100.0 | 77.0 | 100.0 | 100.0 | 77.0 |
|  | LD | 100.0 | 100.0 | 100.0 | 100.0 | 100.0 |
| 28 | LB+ | 98.50 | 73.00 | 98.00 | 100.0 | 70.5 |
|  | LD | 100.0 | 100.0 | 100.0 | 100.0 | 100.0 |
| 29 | LB+ | 96.50 | 63.00 | 95.50 | 100.0 | 57.0 |
|  | LD | 100.0 | 100.0 | 100.0 | 100.0 | 100.0 |
| 30 | LB+ | 98.5 | 69.5 | 95.5 | 98.5 | 62.5 |
|  | LD | 100.0 | 100.0 | 100.0 | 100.0 | 100.0 |
| 31 | LB+ | 93.5 | 69.0 | 94.0 | 99.5 | 59.0 |
|  | LD | 100.0 | 100.0 | 100.0 | 100.0 | 100.0 |
| 32 | LB+ | 92.5 | 62.0 | 90.0 | 99.5 | 48.5 |
|  | LD | 100.0 | 100.0 | 100.0 | 100.0 | 100.0 |
| 33 | LB+ | 90.5 | 60.0 | 95.0 | 99.5 | 48.5 |
|  | LD | 100.0 | 100.0 | 100.0 | 100.0 | 100.0 |
| 34 | LB+ | 89.50 | 57.00 | 94.00 | 100.0 | 44.5 |
|  | LD | 100.0 | 100.0 | 100.0 | 100.0 | 100.0 |
| 35 | LB+ | 88.0 | 62.5 | 95.5 | 100.0 | 49.5 |
|  | LD | 100.0 | 100.0 | 100.0 | 100.0 | 100.0 |
| 36 | LB+ | 86.0 | 53.5 | 90.5 | 100.0 | 36.0 |
|  | LD | 100.0 | 100.0 | 100.0 | 100.0 | 100.0 |
| 37 | LB+ | 80.5 | 55.0 | 85.0 | 100.0 | 32.0 |
|  | LD | 100.0 | 100.0 | 100.0 | 100.0 | 100.0 |
| 38 | LB+ | 84.5 | 58.5 | 84.5 | 100.0 | 36.5 |
|  | LD | 100.0 | 100.0 | 100.0 | 100.0 | 100.0 |
| 39 | LB+ | 77.0 | 57.0 | 82.5 | 100.0 | 30.5 |
|  | LD | 100.0 | 100.0 | 100.0 | 100.0 | 100.0 |
| 40 | LB+ | 76.0 | 63.5 | 64.5 | 100.0 | 20.0 |
|  | LD | 98.0 | 100.0 | 100.0 | 100.0 | 98.0 |
| 41 | LB+ | 72.0 | 56.5 | 78.0 | 100.0 | 20.5 |
|  | LD | 99.5 | 100.0 | 100.0 | 100.0 | 99.5 |
| 42 | LB+ | 74.5 | 36.0 | 86.0 | 100.0 | 18.0 |
|  | LD | 95.5 | 100.0 | 100.0 | 100.0 | 95.5 |
| 43 | LB+ | 65.0 | 28.0 | 90.0 | 99.5 | 12.0 |
|  | LD | 97.5 | 100.0 | 100.0 | 100.0 | 97.5 |
| 44 | LB+ | 66.5 | 29.0 | 89.5 | 100.0 | 12.0 |
|  | LD | 97.5 | 100.0 | 100.0 | 100.0 | 97.5 |
| 45 | LB+ | 61.5 | 20.5 | 86.5 | 99.5 | 4.5 |
|  | LD | 97.0 | 100.0 | 100.0 | 100.0 | 97.0 |
| 46 | LB+ | 64.5 | 19.5 | 87.5 | 99.0 | 7.0 |
|  | LD | 98.5 | 100.0 | 100.0 | 100.0 | 98.5 |
| 47 | LB+ | 48.0 | 13.0 | 87.0 | 99.0 | 2.0 |
|  | LD | 95.5 | 100.0 | 100.0 | 100.0 | 95.5 |
| 48 | LB+ | 45.0 | 8.0 | 92.0 | 99.5 | 2.0 |
|  | LD | 97.0 | 100.0 | 100.0 | 100.0 | 97.0 |
| 49 | LB+ | 48.0 | 10.0 | 86.5 | 99.5 | 1.5 |
|  | LD | 97.0 | 100.0 | 100.0 | 100.0 | 97.0 |
| 50 | LB+ | 39.5 | 3.5 | 89.5 | 99.5 | 0.5 |
|  | LD | 96.0 | 100.0 | 100.0 | 100.0 | 96.0 |
| 51 | LB+ | 43.5 | 5.0 | 89.0 | 98.0 | 1.5 |
|  | LD | 94.0 | 100.0 | 100.0 | 100.0 | 94.0 |
| 52 | LB+ | 43.5 | 3.5 | 92.0 | 99.0 | 1.5 |
|  | LD | 95.5 | 100.0 | 100.0 | 100.0 | 95.5 |
| 53 | LB+ | 39.0 | 4.0 | 88.0 | 97.5 | 0.5 |
|  | LD | 97.5 | 100.0 | 100.0 | 100.0 | 97.5 |
| 54 | LB+ | 45.5 | 5.5 | 90.0 | 98.0 | 1.5 |
|  | LD | 95.5 | 100.0 | 100.0 | 100.0 | 95.5 |
| 55 | LB+ | 42.0 | 4.5 | 92 | 97.5 | 0.5 |
|  | LD | 97.5 | 99.5 | 100.0 | 100.0 | 97.0 |
| 56 | LB+ | 44.5 | 2.5 | 94.0 | 98.5 | 0.0 |
|  | LD | 96.5 | 100.0 | 100.0 | 100.0 | 96.5 |
| 57 | LB+ | 50.5 | 4.0 | 93.5 | 96.5 | 1.0 |
|  | LD | 96.0 | 100.0 | 100.0 | 100.0 | 96.0 |
| 58 | LB+ | 58.0 | 4.0 | 95.0 | 96.0 | 1.5 |
|  | LD | 96.5 | 100.0 | 100.0 | 100.0 | 96.5 |
| 59 | LB+ | 55.0 | 2.5 | 95.0 | 94.0 | 1.5 |
|  | LD | 96.0 | 100.0 | 100.0 | 100.0 | 96.0 |
| 60 | LB+ | 52.0 | 1.5 | 87.0 | 94.5 | 1.5 |
|  | LD | 95.5 | 100.0 | 100.0 | 100.0 | 95.5 |
| 61 | LB+ | 40.0 | 1.5 | 74.0 | 92.5 | 1.0 |
|  | LD | 91.0 | 100.0 | 100.0 | 100.0 | 91.0 |
| 62 | LB+ | 43.0 | 2.0 | 70.0 | 91.0 | 1.5 |
|  | LD | 94.5 | 100.0 | 100.0 | 100.0 | 94.5 |
| 63 | LB+ | 36.0 | 2.5 | 57.5 | 85.0 | 1.5 |
|  | LD | 94.0 | 100.0 | 100.0 | 100.0 | 94.0 |
| 64 | LB+ | 38.5 | 2.0 | 64.0 | 87.0 | 1.5 |
|  | LD | 93.5 | 100.0 | 100.0 | 99.5 | 93.0 |
| 65 | LB+ | 33.0 | 1.00 | 57.0 | 88.5 | 0.5 |
|  | LD | 92.5 | 100.0 | 100.0 | 99.5 | 92.0 |
| 66 | LB+ | 34.5 | 2.0 | 53.5 | 87.0 | 1.0 |
|  | LD | 96.0 | 100.0 | 100.0 | 100.0 | 96.0 |
| 67 | LB+ | 32.5 | 2.5 | 64.5 | 87.0 | 1.0 |
|  | LD | 96.0 | 100.0 | 100.0 | 100.0 | 96.0 |
| 68 | LB+ | 41.0 | 1.5 | 47.0 | 78.5 | 1.0 |
|  | LD | 96.0 | 100.0 | 100.0 | 100.0 | 96.0 |
| 69 | LB+ | 28.5 | 2.5 | 51.5 | 78.5 | 0.5 |
|  | LD | 95.5 | 100.0 | 100.0 | 100.0 | 95.5 |
| 70 | LB+ | 28.0 | 2.0 | 41.5 | 71.5 | 0.5 |
|  | LD | 97.0 | 100.0 | 100.0 | 100.0 | 97.0 |
| 71 | LB+ | 32.0 | 0.5 | 28.5 | 77.5 | 0.5 |
|  | LD | 95.5 | 100.0 | 100.0 | 100.0 | 95.5 |

**Table S2.** Proportions of LB+ and LD hens (*n* = 200/hybrid strain) from batch 2 with intact plumage for four body regions, and the whole body, respectively.

| **Week of Life** | **Hybrid** | **Body Region** | | | | **Whole Body Intact** |
| --- | --- | --- | --- | --- | --- | --- |
|  |  | **Head/neck** | **Back** | **Tail** | **Wing** |  |
| 20 | LB+ | 100.0 | 100.0 | 100.0 | 100.0 | 100.0 |
|  | LD | 100.0 | 100.0 | 100.0 | 100.0 | 100.0 |
| 21 | LB+ | 100.0 | 100.0 | 100.0 | 100.0 | 100.0 |
|  | LD | 100.0 | 100.0 | 100.0 | 100.0 | 100.0 |
| 22 | LB+ | 100.0 | 100.0 | 100.0 | 100.0 | 100.0 |
|  | LD | 100.0 | 100.0 | 100.0 | 100.0 | 100.0 |
| 23 | LB+ | 100.0 | 96.5 | 100.0 | 100.0 | 96.5 |
|  | LD | 100.0 | 100.0 | 100.0 | 100.0 | 100.0 |
| 24 | LB+ | 100.0 | 95.0 | 100.0 | 100.0 | 95.0 |
|  | LD | 100.0 | 100.0 | 100.0 | 100.0 | 100.0 |
| 25 | LB+ | 100.0 | 90.0 | 100.0 | 100.0 | 90.0 |
|  | LD | 100.0 | 100.0 | 100.0 | 100.0 | 100.0 |
| 26 | LB+ | 100.0 | 88.0 | 100.0 | 100.0 | 88.0 |
|  | LD | 100.0 | 100.0 | 100.0 | 100.0 | 100.0 |
| 27 | LB+ | 100.0 | 74.5 | 100.0 | 100.0 | 72.5 |
|  | LD | 100.0 | 100.0 | 100.0 | 100.0 | 100.0 |
| 28 | LB+ | 98.5 | 68.0 | 100.0 | 100.0 | 67.5 |
|  | LD | 100.0 | 100.0 | 100.0 | 100.0 | 100.0 |
| 29 | LB+ | 97.5 | 60.0 | 99.5 | 100.0 | 59.0 |
|  | LD | 100.0 | 100.0 | 100.0 | 100.0 | 100.0 |
| 30 | LB+ | 94.0 | 48.5 | 100.0 | 100.0 | 47.0 |
|  | LD | 100.0 | 100.0 | 100.0 | 100.0 | 100.0 |
| 31 | LB+ | 94.5 | 53.0 | 100.0 | 100.0 | 52.0 |
|  | LD | 100.0 | 100.0 | 100.0 | 100.0 | 100.0 |
| 32 | LB+ | 86.0 | 42.0 | 100.0 | 100.0 | 39.5 |
|  | LD | 100.0 | 100.0 | 100.0 | 100.0 | 100.0 |
| 33 | LB+ | 84.0 | 43.0 | 99.0 | 100.0 | 39.5 |
|  | LD | 100.0 | 100.0 | 100.0 | 100.0 | 100.0 |
| 34 | LB+ | 81.5 | 40.5 | 97.0 | 100.0 | 36.0 |
|  | LD | 100.0 | 100.0 | 100.0 | 100.0 | 100.0 |
| 35 | LB+ | 75.5 | 38.0 | 90.0 | 100.0 | 33.5 |
|  | LD | 100.0 | 100.0 | 100.0 | 100.0 | 100.0 |
| 36 | LB+ | 79.0 | 29.5 | 90.5 | 100.0 | 28.0 |
|  | LD | 100.0 | 100.0 | 100.0 | 100.0 | 100.0 |
| 37 | LB+ | 69.0 | 28.5 | 84.5 | 99.5 | 25.5 |
|  | LD | 100.0 | 100.0 | 100.0 | 100.0 | 100.0 |
| 38 | LB+ | 49.0 | 20.5 | 81.0 | 99.5 | 17.5 |
|  | LD | 100.0 | 100.0 | 100.0 | 100.0 | 100.0 |
| 39 | LB+ | 52.5 | 22.0 | 78.5 | 100.0 | 18.0 |
|  | LD | 100.0 | 100.0 | 100.0 | 100.0 | 100.0 |
| 40 | LB+ | 52.5 | 15.0 | 68.0 | 99.0 | 10.0 |
|  | LD | 100.0 | 100.0 | 100. | 100.0 | 100.0 |
| 41 | LB+ | 45.5 | 16.0 | 66.5 | 98.5 | 13.0 |
|  | LD | 99.0 | 100.0 | 100.0 | 100.0 | 99.0 |
| 42 | LB+ | 34.5 | 16.5 | 67.5 | 97.0 | 8.5 |
|  | LD | 99.5 | 100.0 | 100.0 | 100.0 | 99.5 |
| 43 | LB+ | 41.0 | 13.5 | 61.5 | 96.0 | 10.0 |
|  | LD | 98.5 | 100.0 | 100.0 | 100.0 | 98.5 |
| 44 | LB+ | 41.0 | 11.5 | 48.5 | 93.0 | 7.5 |
|  | LD | 99.0 | 100.0 | 100.0 | 100.0 | 99.0 |
| 45 | LB+ | 39.0 | 10.0 | 56.5 | 95.0 | 7.5 |
|  | LD | 98.5 | 100.0 | 100.0 | 100.0 | 98.5 |
| 46 | LB+ | 41.0 | 6.5 | 52.5 | 90.5 | 5.0 |
|  | LD | 99.0 | 100.0 | 100.0 | 100.0 | 99.0 |
| 48 | LB+ | 37.0 | 8.0 | 34.5 | 89.0 | 6.5 |
|  | LD | 99.0 | 100.0 | 100.0 | 100.0 | 98.0 |
| 49 | LB+ | 35.5 | 9.5 | 36.0 | 89.5 | 7.5 |
|  | LD | 99.0 | 100.0 | 100.0 | 100.0 | 99.0 |
| 50 | LB+ | 38.5 | 6.5 | 34.5 | 86.5 | 4.0 |
|  | LD | 98.0 | 100.0 | 100.0 | 100.0 | 98.0 |
| 51 | LB+ | 29.0 | 7.0 | 29.5 | 85.5 | 4.5 |
|  | LD | 99.0 | 100.0 | 100.0 | 100.0 | 99.0 |
| 52 | LB+ | 27.0 | 9.0 | 30.5 | 80.0 | 6.5 |
|  | LD | 99.5 | 100.0 | 100.0 | 100.0 | 99.5 |
| 53 | LB+ | 35.5 | 6.0 | 27.0 | 84.0 | 6.0 |
|  | LD | 99.0 | 100.0 | 100.0 | 100.0 | 99.0 |
| 54 | LB+ | 23.5 | 5.0 | 19.5 | 78.5 | 4.5 |
|  | LD | 97.5 | 100.0 | 100.0 | 100.0 | 97.5 |
| 55 | LB+ | 24.0 | 5.0 | 23.5 | 78.5 | 3.0 |
|  | LD | 97.0 | 100.0 | 100.0 | 100.0 | 97.0 |
| 56 | LB+ | 27.0 | 7.0 | 20.5 | 68.0 | 6.0 |
|  | LD | 96.5 | 100.0 | 100.0 | 100.0 | 96.5 |
| 57 | LB+ | 29.5 | 4.0 | 17.0 | 71.0 | 3.5 |
|  | LD | 97.5 | 100.0 | 100.0 | 100.0 | 97.5 |
| 58 | LB+ | 21.5 | 5.0 | 15.5 | 86.0 | 4.0 |
|  | LD | 98.0 | 100.0 | 100.0 | 100.0 | 98.0 |
| 60 | LB+ | 28.5 | 6.0 | 12.5 | 74.5 | 3.0 |
|  | LD | 96.0 | 100.0 | 100.0 | 100.0 | 96.0 |
| 62 | LB+ | 28.0 | 3.0 | 11.0 | 74.5 | 2.5 |
|  | LD | 95.5 | 100.0 | 100.0 | 100.0 | 95.5 |
| 63 | LB+ | 24.0 | 2.0 | 8.0 | 78.5 | 1.5 |
|  | LD | 97.0 | 100.0 | 100.0 | 99.5 | 96.5 |
| 64 | LB+ | 29.0 | 4.0 | 9.0 | 72.0 | 4.0 |
|  | LD | 95.0 | 100.0 | 100.0 | 100.0 | 95.0 |
| 65 | LB+ | 18.5 | 1.5 | 6.5 | 72.0 | 1.0 |
|  | LD | 96.0 | 100.0 | 100.0 | 100.0 | 96.0 |
| 66 | LB+ | 20.0 | 2.5 | 13.5 | 72.5 | 2.0 |
|  | LD | 96.0 | 100.0 | 100.0 | 100.0 | 96.0 |
| 67 | LB+ | 21.0 | 5.0 | 8.0 | 73.0 | 3.5 |
|  | LD | 95.5 | 100.0 | 100.0 | 100.0 | 95.5 |
| 68 | LB+ | 24.0 | 2.0 | 8.5 | 69.5 | 2.0 |
|  | LD | 96.0 | 100.0 | 100.0 | 100.0 | 96.0 |
| 69 | LB+ | 23.0 | 3.5 | 10.0 | 69.0 | 2.0 |
|  | LD | 95.5 | 100.0 | 100.0 | 100.0 | 95.5 |

**Table S3.** Proportions of LB+ and LD hens (*n* = 200/hybrid strain) from batch 3 with intact plumage for four body regions, and the whole body, respectively.

| **Week of Life** | **Hybrid** | **Body Region** | | | | **Whole Body Intact** |
| --- | --- | --- | --- | --- | --- | --- |
|  |  | **Head/neck** | **Back** | **Tail** | **Wing** |  |
| 20 | LB+ | 100.0 | 100.0 | 100.0 | 100.0 | 100.0 |
|  | LD | 100.0 | 100.0 | 100.0 | 100.0 | 100.0 |
| 21 | LB+ | 100.0 | 100.0 | 100.0 | 100.0 | 100.0 |
|  | LD | 100.0 | 100.0 | 100.0 | 100.0 | 100.0 |
| 22 | LB+ | 100.0 | 100.0 | 100.0 | 100.0 | 100.0 |
|  | LD | 100.0 | 100.0 | 100.0 | 100.0 | 100.0 |
| 23 | LB+ | 100.0 | 100.0 | 100.0 | 100.0 | 100.0 |
|  | LD | 100.0 | 100.0 | 100.0 | 100.0 | 100.0 |
| 24 | LB+ | 95.5 | 100.0 | 100.0 | 100.0 | 95.5 |
|  | LD | 100.0 | 100.0 | 100.0 | 100.0 | 100.0 |
| 30 | LB+ | 86.5 | 41.0 | 99.0 | 100.0 | 37.0 |
|  | LD | 100.0 | 100.0 | 100.0 | 100.0 | 100.0 |
| 31 | LB+ | 81.0 | 36.0 | 98.0 | 100.0 | 33.0 |
|  | LD | 100.0 | 100.0 | 100.0 | 100.0 | 100.0 |
| 32 | LB+ | 82.0 | 31.5 | 97.0 | 100.0 | 29.5 |
|  | LD | 100.0 | 100.0 | 100.0 | 100.0 | 100.0 |
| 33 | LB+ | 73.0 | 20.5 | 94.5 | 100.0 | 19.5 |
|  | LD | 100.0 | 100.0 | 100.0 | 100.0 | 100.0 |
| 34 | LB+ | 63.0 | 20.0 | 94.5 | 100.0 | 16.0 |
|  | LD | 99.5 | 100.0 | 100.0 | 100.0 | 99.5 |
| 35 | LB+ | 61.5 | 19.0 | 91.0 | 100.0 | 14.5 |
|  | LD | 100.0 | 100.0 | 100.0 | 100.0 | 100.0 |
| 36 | LB+ | 44.0 | 11.5 | 78.5 | 100.0 | 10.0 |
|  | LD | 100.0 | 100.0 | 100.0 | 100.0 | 100.0 |
| 37 | LB+ | 43.0 | 11.0 | 78.0 | 100.0 | 8.5 |
|  | LD | 99.5 | 100.0 | 100.0 | 100.0 | 99.5 |
| 38 | LB+ | 43.5 | 10.0 | 82.0 | 100.0 | 8.5 |
|  | LD | 99.5 | 100.0 | 100.0 | 100.0 | 99.5 |
| 39 | LB+ | 34.5 | 10.5 | 69.5 | 100.0 | 8.0 |
|  | LD | 99.0 | 100.0 | 100.0 | 100.0 | 99.0 |
| 40 | LB+ | 34.0 | 6.5 | 64.5 | 99.5 | 2.5 |
|  | LD | 98.0 | 100.0 | 100.0 | 100.0 | 98.0 |
| 41 | LB+ | 37.0 | 8.5 | 56.0 | 99.5 | 5.5 |
|  | LD | 99.0 | 100.0 | 100.0 | 100.0 | 99.0 |
| 42 | LB+ | 32.0 | 5.5 | 47.0 | 99.5 | 3.5 |
|  | LD | 98.5 | 100.0 | 100.0 | 100.0 | 98.5 |
| 43 | LB+ | 34.5 | 4.0 | 41.5 | 98.0 | 3.0 |
|  | LD | 98.0 | 100.0 | 100.0 | 100.0 | 98.0 |
| 44 | LB+ | 35.5 | 4.0 | 41.0 | 97.5 | 2.0 |
|  | LD | 97.0 | 100.0 | 100.0 | 100.0 | 97.0 |
| 45 | LB+ | 31.0 | 3.5 | 34.5 | 96.5 | 2.0 |
|  | LD | 97.5 | 100.0 | 100.0 | 100.0 | 97.5 |
| 48 | LB+ | 29.5 | 3.0 | 23.0 | 93.0 | 1.5 |
|  | LD | 96.0 | 100.0 | 100.0 | 100.0 | 96.0 |
| 49 | LB+ | 21.0 | 2.5 | 17.5 | 87.5 | 2.0 |
|  | LD | 96.5 | 100.0 | 100.0 | 100.0 | 96.5 |
| 50 | LB+ | 26.0 | 1.5 | 20.0 | 85.5 | 1.0 |
|  | LD | 96.5 | 100.0 | 100.0 | 100.0 | 96.5 |
| 56 | LB+ | 23.5 | 4.5 | 12.5 | 82.5 | 1.5 |
|  | LD | 97.5 | 100.0 | 100.0 | 100.0 | 97.5 |

**Table S4.** Proportions of LB+ and LD hens (*n* = 200/hybrid strain) from batch 1 with moderate plumage loss for four body regions. Min. one body region affected: proportion of hens with at least one body region affected by moderate plumage loss.

| **Week of Life** | **Hybrid** | **Body Region** | | | | **Min. one body region affected** |
| --- | --- | --- | --- | --- | --- | --- |
|  |  | **Head/neck** | **Back** | **Tail** | **Wing** |  |
| 20 | LB+ | 0.0 | 0.0 | 0.0 | 0.0 | 0.0 |
|  | LD | 0.0 | 0.0 | 0.0 | 0.0 | 0.0 |
| 21 | LB+ | 0.0 | 0.0 | 0.0 | 0.0 | 0.0 |
|  | LD | 0.0 | 0.0 | 0.0 | 0.0 | 0.0 |
| 22 | LB+ | 0.0 | 0.0 | 0.0 | 0.0 | 0.0 |
|  | LD | 0.0 | 0.0 | 0.0 | 0.0 | 0.0 |
| 23 | LB+ | 0.0 | 0.0 | 0.0 | 0.0 | 0.0 |
|  | LD | 0.0 | 0.0 | 0.0 | 0.0 | 0.0 |
| 24 | LB+ | 0.0 | 0.0 | 0.0 | 0.0 | 0.0 |
|  | LD | 0.0 | 0.0 | 0.0 | 0.0 | 0.0 |
| 25 | LB+ | 0.0 | 3.0 | 0.0 | 0.0 | 3.0 |
|  | LD | 0.0 | 0.0 | 0.0 | 0.0 | 0.0 |
| 26 | LB+ | 0.0 | 15.5 | 0.0 | 0.0 | 15.5 |
|  | LD | 0.0 | 0.0 | 0.0 | 0.0 | 0.0 |
| 27 | LB+ | 0.0 | 23.0 | 0.0 | 0.0 | 23.0 |
|  | LD | 0.0 | 0.0 | 0.0 | 0.0 | 0.0 |
| 28 | LB+ | 1.5 | 27.0 | 2.0 | 0.0 | 29.0 |
|  | LD | 0.0 | 0.0 | 0.0 | 0.0 | 0.0 |
| 29 | LB+ | 3.5 | 37.0 | 4.5 | 0.0 | 43.0 |
|  | LD | 0.0 | 0.0 | 0.0 | 0.0 | 0.0 |
| 30 | LB+ | 1.5 | 30.5 | 4.5 | 1.5 | 37.5 |
|  | LD | 0.0 | 0.0 | 0.0 | 0.0 | 0.0 |
| 31 | LB+ | 6.5 | 31.0 | 6.0 | 0.5 | 41.0 |
|  | LD | 0.0 | 0.0 | 0.0 | 0.0 | 0.0 |
| 32 | LB+ | 7.5 | 38.0 | 10.0 | 0.5 | 51.0 |
|  | LD | 0.0 | 0.0 | 0.0 | 0.0 | 0.0 |
| 33 | LB+ | 9.5 | 40.0 | 5.0 | 0.5 | 51.5 |
|  | LD | 0.0 | 0.0 | 0.0 | 0.0 | 0.0 |
| 34 | LB+ | 10.5 | 43.0 | 6.0 | 0.0 | 55.5 |
|  | LD | 0.0 | 0.0 | 0.0 | 0.0 | 0.0 |
| 35 | LB+ | 12.0 | 37.5 | 4.5 | 0.0 | 49.5 |
|  | LD | 0.0 | 0.0 | 0.0 | 0.0 | 0.0 |
| 36 | LB+ | 14.0 | 46.5 | 9.5 | 0.0 | 64.0 |
|  | LD | 0.0 | 0.0 | 0.0 | 0.0 | 0.0 |
| 37 | LB+ | 19.5 | 45.0 | 15.0 | 0.0 | 67.5 |
|  | LD | 0.0 | 0.0 | 0.0 | 0.0 | 0.0 |
| 38 | LB+ | 15.5 | 41.5 | 15.5 | 0.0 | 64.0 |
|  | LD | 0.0 | 0.0 | 0.0 | 0.0 | 0.0 |
| 39 | LB+ | 23.0 | 43.0 | 17.5 | 0.0 | 69.5 |
|  | LD | 0.0 | 0.0 | 0.0 | 0.0 | 0.0 |
| 40 | LB+ | 24.0 | 36.5 | 35.5 | 0.0 | 80.0 |
|  | LD | 2.0 | 0.0 | 0.0 | 0.0 | 2.0 |
| 41 | LB+ | 28.0 | 43.5 | 22.0 | 0.0 | 77.5 |
|  | LD | 0.50 | 0.0 | 0.0 | 0.0 | 0.5 |
| 42 | LB+ | 25.5 | 64.0 | 14.0 | 0.0 | 82.0 |
|  | LD | 4.5 | 0.0 | 0.0 | 0.0 | 4.5 |
| 43 | LB+ | 35.0 | 72.0 | 10.0 | 0.5 | 88.0 |
|  | LD | 2.5 | 0.0 | 0.0 | 0.0 | 2.5 |
| 44 | LB+ | 33.5 | 71.0 | 10.5 | 0.0 | 88.0 |
|  | LD | 2.5 | 0.0 | 0.0 | 0.0 | 2.5 |
| 45 | LB+ | 38.5 | 78.0 | 13.5 | 0.5 | 91.5 |
|  | LD | 3.0 | 0.0 | 0.0 | 0.0 | 3.0 |
| 46 | LB+ | 35.5 | 80.0 | 12.5 | 1.0 | 92.0 |
|  | LD | 1.5 | 0.0 | 0.0 | 0.0 | 1.5 |
| 47 | LB+ | 52.0 | 84.0 | 13.0 | 1.0 | 95.0 |
|  | LD | 4.5 | 0.0 | 0.0 | 0.0 | 4.5 |
| 48 | LB+ | 55.0 | 84.5 | 8.0 | 0.5 | 95.0 |
|  | LD | 3.0 | 0.0 | 0.0 | 0.0 | 3.0 |
| 49 | LB+ | 52.0 | 82.5 | 13.5 | 0.5 | 95.0 |
|  | LD | 3.0 | 0.0 | 0.0 | 0.0 | 3.0 |
| 50 | LB+ | 60.5 | 80.5 | 10.5 | 0.5 | 92.0 |
|  | LD | 4.0 | 0.0 | 0.0 | 0.0 | 4.0 |
| 51 | LB+ | 56.5 | 74.0 | 11.0 | 2.0 | 90.0 |
|  | LD | 6.0 | 0.0 | 0.0 | 0.0 | 6.0 |
| 52 | LB+ | 56.5 | 74.0 | 8.0 | 1.0 | 88.0 |
|  | LD | 4.5 | 0.0 | 0.0 | 0.0 | 4.5 |
| 53 | LB+ | 61.0 | 69.0 | 12.0 | 2.5 | 90.5 |
|  | LD | 2.5 | 0.0 | 0.0 | 0.0 | 2.5 |
| 54 | LB+ | 54.5 | 63.0 | 10.0 | 2.0 | 84.0 |
|  | LD | 4.5 | 0.0 | 0.0 | 0.0 | 4.5 |
| 55 | LB+ | 58.0 | 63.5 | 8.0 | 2.5 | 86.5 |
|  | LD | 2.5 | 0.0 | 0.0 | 0.0 | 2.5 |
| 56 | LB+ | 55.5 | 59.0 | 6.0 | 1.5 | 83.5 |
|  | LD | 3.5 | 0.0 | 0.0 | 0.0 | 3.5 |
| 57 | LB+ | 49.5 | 54.0 | 6.5 | 3.5 | 81.0 |
|  | LD | 4.0 | 0.0 | 0.0 | 0.0 | 4.0 |
| 58 | LB+ | 42.0 | 57.0 | 5.0 | 4.0 | 80.5 |
|  | LD | 3.5 | 0.0 | 0.0 | 0.0 | 3.5 |
| 59 | LB+ | 45.0 | 54.5 | 5.0 | 6.0 | 78.0 |
|  | LD | 4.0 | 0.0 | 0.0 | 0.0 | 4.0 |
| 60 | LB+ | 48.0 | 43.0 | 13.0 | 5.5 | 78.0 |
|  | LD | 4.5 | 0.0 | 0.0 | 0.0 | 4.5 |
| 61 | LB+ | 60.0 | 34.0 | 27.0 | 7.5 | 80.5 |
|  | LD | 9.0 | 0.0 | 0.0 | 0.0 | 9.0 |
| 62 | LB+ | 57.0 | 29.5 | 29.5 | 9.0 | 82.5 |
|  | LD | 5.5 | 0.0 | 0.0 | 0.0 | 5.5 |
| 63 | LB+ | 63.5 | 31.0 | 28.5 | 14.5 | 84.0 |
|  | LD | 6.0 | 0.0 | 0.0 | 0.0 | 6.0 |
| 64 | LB+ | 61.5 | 29.5 | 34.0 | 13.0 | 84.0 |
|  | LD | 6.5 | 0.0 | 0.0 | 0.0 | 6.5 |
| 65 | LB+ | 67.0 | 27.5 | 41.5 | 11.5 | 87.0 |
|  | LD | 7.5 | 0.0 | 0.0 | 0.0 | 7.5 |
| 66 | LB+ | 65.0 | 30.5 | 43.5 | 13.0 | 87.5 |
|  | LD | 4.0 | 0.0 | 0.0 | 0.0 | 4.0 |
| 67 | LB+ | 66.5 | 32.0 | 37.0 | 13.0 | 86.5 |
|  | LD | 4.0 | 0.0 | 0.0 | 0.0 | 4.0 |
| 68 | LB+ | 59.0 | 22.0 | 50.5 | 21.5 | 87.5 |
|  | LD | 4.0 | 0.0 | 0.0 | 0.0 | 4.0 |
| 69 | LB+ | 71.5 | 25.0 | 45.5 | 21.5 | 89.0 |
|  | LD | 4.5 | 0.0 | 0.0 | 0.0 | 4.5 |
| 70 | LB+ | 70.0 | 26.5 | 53.5 | 28.5 | 93.0 |
|  | LD | 3.0 | 0.0 | 0.0 | 0.0 | 3.0 |
| 71 | LB+ | 68.0 | 26.5 | 63.0 | 22.5 | 97.5 |
|  | LD | 4.5 | 0.0 | 0.0 | 0.0 | 4.5 |

**Table S5.** Proportions of LB+ and LD hens (*n* = 200/hybrid strain) from batch 2 with moderate plumage loss for four body regions. Min. one body region affected: proportion of hens with at least one body region affected by moderate plumage loss.

| **Week of Life** | **Hybrid** | **Body Region** | | | | **Min. one body region affected** |
| --- | --- | --- | --- | --- | --- | --- |
|  |  | **Head/neck** | **Back** | **Tail** | **Wing** |  |
| 20 | LB+ | 0.0 | 0.0 | 0.0 | 0.0 | 0.0 |
|  | LD | 0.0 | 0.0 | 0.0 | 0.0 | 0.0 |
| 21 | LB+ | 0.0 | 0.0 | 0.0 | 0.0 | 0.0 |
|  | LD | 0.0 | 0.0 | 0.0 | 0.0 | 0.0 |
| 22 | LB+ | 0.0 | 0.0 | 0.0 | 0.0 | 0.0 |
|  | LD | 0.0 | 0.0 | 0.0 | 0.0 | 0.0 |
| 23 | LB+ | 0.0 | 3.0 | 0.0 | 0.0 | 3.5 |
|  | LD | 0.0 | 0.0 | 0.0 | 0.0 | 0.0 |
| 24 | LB+ | 0.0 | 5.0 | 0.0 | 0.0 | 5.0 |
|  | LD | 0.0 | 0.0 | 0.0 | 0.0 | 0.0 |
| 25 | LB+ | 0.0 | 10.0 | 0.0 | 0.0 | 10.0 |
|  | LD | 0.0 | 0.0 | 0.0 | 0.0 | 0.0 |
| 26 | LB+ | 0.0 | 12.0 | 0.0 | 0.0 | 12.0 |
|  | LD | 0.0 | 0.0 | 0.0 | 0.0 | 0.0 |
| 27 | LB+ | 0.0 | 25.5 | 0.0 | 0.0 | 25.5 |
|  | LD | 0.0 | 0.0 | 0.0 | 0.0 | 0.0 |
| 28 | LB+ | 0.0 | 32.0 | 0.0 | 0.0 | 32.0 |
|  | LD | 0.0 | 0.0 | 0.0 | 0.0 | 0.0 |
| 29 | LB+ | 0.0 | 40.0 | 0.0 | 0.0 | 40.0 |
|  | LD | 0.0 | 0.0 | 0.0 | 0.0 | 0.0 |
| 30 | LB+ | 6.0 | 51.5 | 0.0 | 0.0 | 53.0 |
|  | LD | 0.0 | 0.0 | 0.0 | 0.0 | 0.0 |
| 31 | LB+ | 5.5 | 47.0 | 0.0 | 0.0 | 48.0 |
|  | LD | 0.0 | 0.0 | 0.0 | 0.0 | 0.0 |
| 32 | LB+ | 14.0 | 58.0 | 0.0 | 0.0 | 60.5 |
|  | LD | 0.0 | 0.0 | 0.0 | 0.0 | 0.0 |
| 33 | LB+ | 16.0 | 57.0 | 1.0 | 0.0 | 60.5 |
|  | LD | 0.0 | 0.0 | 0.0 | 0.0 | 0.0 |
| 34 | LB+ | 18.5 | 59.5 | 3.0 | 0.0 | 64.0 |
|  | LD | 0.0 | 0.0 | 0.0 | 0.0 | 0.0 |
| 35 | LB+ | 24.5 | 62.0 | 10.0 | 0.0 | 65.5 |
|  | LD | 0.0 | 0.0 | 0.0 | 0.0 | 0.0 |
| 36 | LB+ | 21.0 | 70.5 | 9.5 | 0.0 | 71.5 |
|  | LD | 0.0 | 0.0 | 0.0 | 0.0 | 0.0 |
| 37 | LB+ | 31.0 | 71.0 | 15.5 | 0.5 | 73.5 |
|  | LD | 0.0 | 0.0 | 0.0 | 0.0 | 0.0 |
| 38 | LB+ | 51.0 | 79.0 | 19.0 | 0.0 | 82.0 |
|  | LD | 0.0 | 0.0 | 0.0 | 0.0 | 0.0 |
| 39 | LB+ | 47.5 | 74.0 | 21.0 | 0.0 | 80.5 |
|  | LD | 0.0 | 0.0 | 0.0 | 0.0 | 0.0 |
| 40 | LB+ | 47.5 | 83.5 | 32.0 | 1.0 | 90.0 |
|  | LD | 0.0 | 0.0 | 0.0 | 0.0 | 0.0 |
| 41 | LB+ | 54.5 | 82.0 | 33.5 | 1.5 | 87.0 |
|  | LD | 1.0 | 0.0 | 0.0 | 0.0 | 1.0 |
| 42 | LB+ | 65.5 | 80.0 | 30.5 | 2.5 | 51.0 |
|  | LD | 0.5 | 0.0 | 0.0 | 0.0 | 0.5 |
| 43 | LB+ | 59.0 | 80.0 | 38.5 | 4.0 | 90.0 |
|  | LD | 1.5 | 0.0 | 0.0 | 0.0 | 1.5 |
| 44 | LB+ | 59.0 | 77.5 | 50.0 | 7.0 | 92.0 |
|  | LD | 1.0 | 0.0 | 0.0 | 0.0 | 1.0 |
| 45 | LB+ | 61.0 | 79.0 | 42.0 | 5.0 | 91.5 |
|  | LD | 1.5 | 0.0 | 0.0 | 0.0 | 1.5 |
| 46 | LB+ | 59.0 | 82.0 | 44.0 | 9.5 | 95.0 |
|  | LD | 1.0 | 0.0 | 0.0 | 0.0 | 1.0 |
| 48 | LB+ | 63.0 | 70.0 | 56.5 | 11.0 | 92.5 |
|  | LD | 1.0 | 0.0 | 0.0 | 0.0 | 1.0 |
| 49 | LB+ | 64.5 | 49.0 | 55.0 | 10.0 | 89.5 |
|  | LD | 1.0 | 0.0 | 0.0 | 0.0 | 1.0 |
| 50 | LB+ | 61.5 | 43.0 | 39.5 | 13.5 | 89.5 |
|  | LD | 2.0 | 0.0 | 0.0 | 0.0 | 2.0 |
| 51 | LB+ | 71.0 | 28.5 | 35.5 | 14.5 | 90.0 |
|  | LD | 1.0 | 0.0 | 0.0 | 0.0 | 1.0 |
| 52 | LB+ | 73.0 | 29.0 | 20.5 | 20.0 | 87.5 |
|  | LD | 0.5 | 0.0 | 0.0 | 0.0 | 0.5 |
| 53 | LB+ | 64.5 | 27.0 | 19.0 | 16.0 | 78.0 |
|  | LD | 1.0 | 0.0 | 0.0 | 0.0 | 1.0 |
| 54 | LB+ | 81.5 | 21.0 | 20.0 | 21.5 | 91.5 |
|  | LD | 2.5 | 0.0 | 0.0 | 0.0 | 2.5 |
| 55 | LB+ | 76.0 | 23.5 | 17.5 | 21.5 | 88.0 |
|  | LD | 3.0 | 0.0 | 0.0 | 0.0 | 3.0 |
| 56 | LB+ | 72.5 | 18.5 | 12.0 | 32.0 | 85.0 |
|  | LD | 3.5 | 0.0 | 0.0 | 0.0 | 3.5 |
| 57 | LB+ | 70.0 | 15.0 | 10.5 | 29.0 | 82.5 |
|  | LD | 2.5 | 0.0 | 0.0 | 0.0 | 2.5 |
| 58 | LB+ | 78.5 | 16.5 | 9.0 | 14.0 | 87.0 |
|  | LD | 2.0 | 0.0 | 0.0 | 0.0 | 2.0 |
| 60 | LB+ | 71.5 | 11.0 | 10.0 | 25.5 | 81.0 |
|  | LD | 4.0 | 0.0 | 0.0 | 0.0 | 4.0 |
| 62 | LB+ | 72.0 | 17.5 | 8.0 | 24.5 | 80.5 |
|  | LD | 4.5 | 0.0 | 0.0 | 0.0 | 4.5 |
| 63 | LB+ | 76.0 | 17.5 | 9.5 | 20.5 | 86.0 |
|  | LD | 3.0 | 0.0 | 0.0 | 0.5 | 3.5 |
| 64 | LB+ | 71.0 | 15.5 | 3.5 | 27.5 | 81.0 |
|  | LD | 5.0 | 0.0 | 0.0 | 0.0 | 5.0 |
| 65 | LB+ | 81.5 | 15.5 | 5.5 | 27.5 | 89.0 |
|  | LD | 4.0 | 0.0 | 0.0 | 0.0 | 4.0 |
| 66 | LB+ | 79.5 | 17.0 | 4.0 | 28.5 | 87.5 |
|  | LD | 4.0 | 0.0 | 0.0 | 0.0 | 4.0 |
| 67 | LB+ | 78.5 | 6.0 | 2.0 | 27.0 | 82.0 |
|  | LD | 5.0 | 0.0 | 0.0 | 0.0 | 5.0 |
| 68 | LB+ | 76.0 | 15.0 | 3.5 | 30.0 | 85.5 |
|  | LD | 4.0 | 0.0 | 0.0 | 0.0 | 4.0 |
| 69 | LB+ | 76.0 | 11.0 | 2.5 | 30.0 | 84.0 |
|  | LD | 4.5 | 0.0 | 0.0 | 0.0 | 4.5 |

**Table S6.** Proportions of LB+ and LD hens (*n* = 200/hybrid strain) from batch 3 with moderate plumage loss for four body regions. Min. one body region affected: proportion of hens with at least one body region affected by moderate plumage loss.

| **Week of Life** | **Hybrid** | **Body Region** | | | | **Min. one body region affected** |
| --- | --- | --- | --- | --- | --- | --- |
|  |  | **Head/neck** | **Back** | **Tail** | **Wing** |  |
| 20 | LB+ | 0.0 | 0.0 | 0.0 | 0.0 | 0.0 |
|  | LD | 0.0 | 0.0 | 0.0 | 0.0 | 0.0 |
| 21 | LB+ | 0.0 | 0.0 | 0.0 | 0.0 | 0.0 |
|  | LD | 0.0 | 0.0 | 0.0 | 0.0 | 0.0 |
| 22 | LB+ | 0.0 | 0.0 | 0.0 | 0.0 | 0.0 |
|  | LD | 0.0 | 0.0 | 0.0 | 0.0 | 0.0 |
| 23 | LB+ | 0.0 | 0.0 | 0.0 | 0.0 | 0.0 |
|  | LD | 0.0 | 0.0 | 0.0 | 0.0 | 0.0 |
| 24 | LB+ | 0.0 | 2.5 | 0.0 | 0.0 | 2.5 |
|  | LD | 0.0 | 0.0 | 0.0 | 0.0 | 0.0 |
| 30 | LB+ | 13.5 | 59.0 | 1.0 | 0.0 | 61.5 |
|  | LD | 0.0 | 0.0 | 0.0 | 0.0 | 0.0 |
| 31 | LB+ | 19.0 | 64.0 | 2.0 | 0.0 | 67.0 |
|  | LD | 0.0 | 0.0 | 0.0 | 0.0 | 0.0 |
| 32 | LB+ | 18.0 | 68.5 | 3.0 | 0.0 | 70.5 |
|  | LD | 0.0 | 0.0 | 0.0 | 0.0 | 0.0 |
| 33 | LB+ | 27.0 | 79.5 | 5.5 | 0.0 | 80.5 |
|  | LD | 0.0 | 0.0 | 0.0 | 0.0 | 0.0 |
| 34 | LB+ | 37.0 | 80.0 | 5.5 | 0.0 | 84.0 |
|  | LD | 0.5 | 0.0 | 0.0 | 0.0 | 0.5 |
| 35 | LB+ | 38.5 | 81.0 | 9.0 | 0.0 | 85.0 |
|  | LD | 0.0 | 0.0 | 0.0 | 0.0 | 0.0 |
| 36 | LB+ | 56.0 | 88.0 | 21.5 | 0.0 | 89.5 |
|  | LD | 0.0 | 0.0 | 0.0 | 0.0 | 0.0 |
| 37 | LB+ | 57.0 | 89.0 | 22.0 | 0.0 | 91.5 |
|  | LD | 0.5 | 0.0 | 0.0 | 0.0 | 0.5 |
| 38 | LB+ | 56.5 | 89.0 | 18.0 | 0.0 | 90.5 |
|  | LD | 0.5 | 0.0 | 0.0 | 0.0 | 0.5 |
| 39 | LB+ | 65.5 | 86.5 | 30.5 | 0.0 | 92.0 |
|  | LD | 1.0 | 0.0 | 0.0 | 0.0 | 1.0 |
| 40 | LB+ | 66.0 | 91.5 | 33.0 | 0.5 | 96.5 |
|  | LD | 2.0 | 0.0 | 0.0 | 0.0 | 2.0 |
| 41 | LB+ | 63.0 | 90.5 | 43.5 | 0.5 | 94.5 |
|  | LD | 1.0 | 0.0 | 0.0 | 0.0 | 1.0 |
| 42 | LB+ | 68.0 | 90.0 | 50.0 | 0.5 | 96.5 |
|  | LD | 1.5 | 0.0 | 0.0 | 0.0 | 1.5 |
| 43 | LB+ | 65.5 | 89.0 | 56.0 | 2.0 | 97.0 |
|  | LD | 2.0 | 0.0 | 0.0 | 0.0 | 2.0 |
| 44 | LB+ | 64.5 | 86.0 | 56.5 | 2.5 | 98.0 |
|  | LD | 3.0 | 0.0 | 0.0 | 0.0 | 3.0 |
| 45 | LB+ | 69.0 | 81.5 | 60.5 | 3.5 | 97.0 |
|  | LD | 2.5 | 0.0 | 0.0 | 0.0 | 2.5 |
| 48 | LB+ | 70.5 | 72.0 | 44.5 | 7.0 | 97.0 |
|  | LD | 4.0 | 0.0 | 0.0 | 0.0 | 4.0 |
| 49 | LB+ | 78.5 | 62.5 | 34.5 | 12.5 | 95.0 |
|  | LD | 3.5 | 0.0 | 0.0 | 0.0 | 3.5 |
| 50 | LB+ | 74.0 | 42.0 | 21.5 | 14.5 | 90.0 |
|  | LD | 3.5 | 0.0 | 0.0 | 0.0 | 3.5 |
| 56 | LB+ | 76.5 | 33.0 | 19.0 | 17.5 | 91.0 |
|  | LD | 2.5 | 0.0 | 0.0 | 0.0 | 2.5 |

**Table S7.** Proportions of LB+ and LD hens (*n* = 200/hybrid strain) from batch 1 with severe plumage loss for four body regions. Min. one body region affected: proportion of hens with at least one body region affected by severe plumage loss.

| **Week of Life** | **Hybrid** | **Body Region** | | | | **Min. one body region affected** |
| --- | --- | --- | --- | --- | --- | --- |
|  |  | **Head/neck** | **Back** | **Tail** | **Wing** |  |
| 20 | LB+ | 0.0 | 0.0 | 0.0 | 0.0 | 0.0 |
|  | LD | 0.0 | 0.0 | 0.0 | 0.0 | 0.0 |
| 21 | LB+ | 0.0 | 0.0 | 0.0 | 0.0 | 0.0 |
|  | LD | 0.0 | 0.0 | 0.0 | 0.0 | 0.0 |
| 22 | LB+ | 0.0 | 0.0 | 0.0 | 0.0 | 0.0 |
|  | LD | 0.0 | 0.0 | 0.0 | 0.0 | 0.0 |
| 23 | LB+ | 0.0 | 0.0 | 0.0 | 0.0 | 0.0 |
|  | LD | 0.0 | 0.0 | 0.0 | 0.0 | 0.0 |
| 24 | LB+ | 0.0 | 0.0 | 0.0 | 0.0 | 0.0 |
|  | LD | 0.0 | 0.0 | 0.0 | 0.0 | 0.0 |
| 25 | LB+ | 0.0 | 0.0 | 0.0 | 0.0 | 0.0 |
|  | LD | 0.0 | 0.0 | 0.0 | 0.0 | 0.0 |
| 26 | LB+ | 0.0 | 0.0 | 0.0 | 0.0 | 0.0 |
|  | LD | 0.0 | 0.0 | 0.0 | 0.0 | 0.0 |
| 27 | LB+ | 0.0 | 0.0 | 0.0 | 0.0 | 0.0 |
|  | LD | 0.0 | 0.0 | 0.0 | 0.0 | 0.0 |
| 28 | LB+ | 0.0 | 0.0 | 0.0 | 0.0 | 0.0 |
|  | LD | 0.0 | 0.0 | 0.0 | 0.0 | 0.0 |
| 29 | LB+ | 0.0 | 0.0 | 0.0 | 0.0 | 0.0 |
|  | LD | 0.0 | 0.0 | 0.0 | 0.0 | 0.0 |
| 30 | LB+ | 0.0 | 0.0 | 0.0 | 0.0 | 0.0 |
|  | LD | 0.0 | 0.0 | 0.0 | 0.0 | 0.0 |
| 31 | LB+ | 0.0 | 0.0 | 0.0 | 0.0 | 0.0 |
|  | LD | 0.0 | 0.0 | 0.0 | 0.0 | 0.0 |
| 32 | LB+ | 0.0 | 0.0 | 0.0 | 0.0 | 0.0 |
|  | LD | 0.0 | 0.0 | 0.0 | 0.0 | 0.0 |
| 33 | LB+ | 0.0 | 0.0 | 0.0 | 0.0 | 0.0 |
|  | LD | 0.0 | 0.0 | 0.0 | 0.0 | 0.0 |
| 34 | LB+ | 0.0 | 0.0 | 0.0 | 0.0 | 0.0 |
|  | LD | 0.0 | 0.0 | 0.0 | 0.0 | 0.0 |
| 35 | LB+ | 0.0 | 0.0 | 0.0 | 0.0 | 0.0 |
|  | LD | 0.0 | 0.0 | 0.0 | 0.0 | 0.0 |
| 36 | LB+ | 0.0 | 0.0 | 0.0 | 0.0 | 0.0 |
|  | LD | 0.0 | 0.0 | 0.0 | 0.0 | 0.0 |
| 37 | LB+ | 0.0 | 0.0 | 0.0 | 0.0 | 0.0 |
|  | LD | 0.0 | 0.0 | 0.0 | 0.0 | 0.0 |
| 38 | LB+ | 0.0 | 0.0 | 0.0 | 0.0 | 0.0 |
|  | LD | 0.0 | 0.0 | 0.0 | 0.0 | 0.0 |
| 39 | LB+ | 0.0 | 0.0 | 0.0 | 0.0 | 0.0 |
|  | LD | 0.0 | 0.0 | 0.0 | 0.0 | 0.0 |
| 40 | LB+ | 0.0 | 0.0 | 0.0 | 0.0 | 0.0 |
|  | LD | 0.0 | 0.0 | 0.0 | 0.0 | 0.0 |
| 41 | LB+ | 0.0 | 0.0 | 0.0 | 0.0 | 0.0 |
|  | LD | 0.0 | 0.0 | 0.0 | 0.0 | 0.0 |
| 42 | LB+ | 0.0 | 0.0 | 0.0 | 0.0 | 0.0 |
|  | LD | 0.0 | 0.0 | 0.0 | 0.0 | 0.0 |
| 43 | LB+ | 0.0 | 0.0 | 0.0 | 0.0 | 0.0 |
|  | LD | 0.0 | 0.0 | 0.0 | 0.0 | 0.0 |
| 44 | LB+ | 0.0 | 0.0 | 0.0 | 0.0 | 0.0 |
|  | LD | 0.0 | 0.0 | 0.0 | 0.0 | 0.0 |
| 45 | LB+ | 0.0 | 1.5 | 0.0 | 0.0 | 1.5 |
|  | LD | 0.0 | 0.0 | 0.0 | 0.0 | 0.0 |
| 46 | LB+ | 0.0 | 0.5 | 0.0 | 0.0 | 0.5 |
|  | LD | 0.0 | 0.0 | 0.0 | 0.0 | 0.0 |
| 47 | LB+ | 0.0 | 3.0 | 0.0 | 0.0 | 3.0 |
|  | LD | 0.0 | 0.0 | 0.0 | 0.0 | 0.0 |
| 48 | LB+ | 0.0 | 7.5 | 0.0 | 0.0 | 7.5 |
|  | LD | 0.0 | 0.0 | 0.0 | 0.0 | 0.0 |
| 49 | LB+ | 0.0 | 7.5 | 0.0 | 0.0 | 7.5 |
|  | LD | 0.0 | 0.0 | 0.0 | 0.0 | 0.0 |
| 50 | LB+ | 0.0 | 16.0 | 0.0 | 0.0 | 16.0 |
|  | LD | 0.0 | 0.0 | 0.0 | 0.0 | 0.0 |
| 51 | LB+ | 0.0 | 21.0 | 0.0 | 0.0 | 21.0 |
|  | LD | 0.0 | 0.0 | 0.0 | 0.0 | 0.0 |
| 52 | LB+ | 0.0 | 22.5 | 0.0 | 0.0 | 22.5 |
|  | LD | 0.0 | 0.0 | 0.0 | 0.0 | 0.0 |
| 53 | LB+ | 0.0 | 27.0 | 0.0 | 0.0 | 27.0 |
|  | LD | 0.0 | 0.0 | 0.0 | 0.0 | 0.0 |
| 54 | LB+ | 0.0 | 31.5 | 0.0 | 0.0 | 31.5 |
|  | LD | 0.0 | 0.0 | 0.0 | 0.0 | 0.0 |
| 55 | LB+ | 0.0 | 32.0 | 0.0 | 0.0 | 32.0 |
|  | LD | 0.0 | 0.0 | 0.0 | 0.0 | 0.0 |
| 56 | LB+ | 0.0 | 38.5 | 0.0 | 0.0 | 38.5 |
|  | LD | 0.0 | 0.0 | 0.0 | 0.0 | 0.0 |
| 57 | LB+ | 0.0 | 42.0 | 0.0 | 0.0 | 42.0 |
|  | LD | 0.0 | 0.0 | 0.0 | 0.0 | 0.0 |
| 58 | LB+ | 0.0 | 39.0 | 0.0 | 0.0 | 39.0 |
|  | LD | 0.0 | 0.0 | 0.0 | 0.0 | 0.0 |
| 59 | LB+ | 0.0 | 43.0 | 0.0 | 0.0 | 43.0 |
|  | LD | 0.0 | 0.0 | 0.0 | 0.0 | 0.0 |
| 60 | LB+ | 0.0 | 55.5 | 0.0 | 0.0 | 55.5 |
|  | LD | 0.0 | 0.0 | 0.0 | 0.0 | 0.0 |
| 61 | LB+ | 0.0 | 64.5 | 0.0 | 0.0 | 64.5 |
|  | LD | 0.0 | 0.0 | 0.0 | 0.0 | 0.0 |
| 62 | LB+ | 0.0 | 68.5 | 0.5 | 0.0 | 68.5 |
|  | LD | 0.0 | 0.0 | 0.0 | 0.0 | 0.0 |
| 63 | LB+ | 0.5 | 66.5 | 2.5 | 0.5 | 67.0 |
|  | LD | 0.0 | 0.0 | 0.0 | 0.0 | 0.0 |
| 64 | LB+ | 0.0 | 68.5 | 2.0 | 0.0 | 68.5 |
|  | LD | 0.0 | 0.0 | 0.0 | 0.0 | 0.0 |
| 65 | LB+ | 0.0 | 71.5 | 1.5 | 0.0 | 71.5 |
|  | LD | 0.0 | 0.0 | 0.0 | 0.0 | 0.0 |
| 66 | LB+ | 0.5 | 67.5 | 3.0 | 0.0 | 68.0 |
|  | LD | 0.0 | 0.0 | 0.0 | 0.0 | 0.0 |
| 67 | LB+ | 0.5 | 65.5 | 1.5 | 0.0 | 65.5 |
|  | LD | 0.0 | 0.0 | 0.0 | 0.0 | 0.0 |
| 68 | LB+ | 0.0 | 76.5 | 2.5 | 0.0 | 76.5 |
|  | LD | 0.0 | 0.0 | 0.0 | 0.0 | 0.0 |
| 69 | LB+ | 0.0 | 72.5 | 3.0 | 0.0 | 72.5 |
|  | LD | 0.0 | 0.0 | 0.0 | 0.0 | 0.0 |
| 70 | LB+ | 2.0 | 71.5 | 5.0 | 0.0 | 72.0 |
|  | LD | 0.0 | 0.0 | 0.0 | 0.0 | 0.0 |
| 71 | LB+ | 0.0 | 73.0 | 8.5 | 0.0 | 73.0 |
|  | LD | 0.0 | 0.0 | 0.0 | 0.0 | 0.0 |

**Table S8.** Proportions of LB+ and LD hens (*n* = 200/hybrid strain) from batch 2 with severe plumage loss for four body regions. Min. one body region affected: proportion of hens with at least one body region affected by severe plumage loss.

| **Week of Life** | **Hybrid** | **Body Region** | | | | **Min. one body region affected** | |
| --- | --- | --- | --- | --- | --- | --- | --- |
|  |  | **Head/neck** | **Back** | **Tail** | **Wing** |  |  |
| 20 | LB+ | 0.0 | 0.0 | 0.0 | 0.0 | 0.0 | |
|  | LD | 0.0 | 0.0 | 0.0 | 0.0 | 0.0 | |
| 21 | LB+ | 0.0 | 0.0 | 0.0 | 0.0 | 0.0 | |
|  | LD | 0.0 | 0.0 | 0.0 | 0.0 | 0.0 | |
| 22 | LB+ | 0.0 | 0.0 | 0.0 | 0.0 | 0.0 | |
|  | LD | 0.0 | 0.0 | 0.0 | 0.0 | 0.0 | |
| 23 | LB+ | 0.0 | 0.0 | 0.0 | 0.0 | 0.0 | |
|  | LD | 0.0 | 0.0 | 0.0 | 0.0 | 0.0 | |
| 24 | LB+ | 0.0 | 0.0 | 0.0 | 0.0 | 0.0 | |
|  | LD | 0.0 | 0.0 | 0.0 | 0.0 | 0.0 | |
| 25 | LB+ | 0.0 | 0.0 | 0.0 | 0.0 | 0.0 | |
|  | LD | 0.0 | 0.0 | 0.0 | 0.0 | 0.0 | |
| 26 | LB+ | 0.0 | 0.0 | 0.0 | 0.0 | 0.0 | |
|  | LD | 0.0 | 0.0 | 0.0 | 0.0 | 0.0 | |
| 27 | LB+ | 0.0 | 0.0 | 0.0 | 0.0 | 0.0 | |
|  | LD | 0.0 | 0.0 | 0.0 | 0.0 | 0.0 | |
| 28 | LB+ | 0.0 | 0.0 | 0.0 | 0.0 | 0.0 | |
|  | LD | 0.0 | 0.0 | 0.0 | 0.0 | 0.0 | |
| 29 | LB+ | 0.0 | 0.0 | 0.0 | 0.0 | 0.0 | |
|  | LD | 0.0 | 0.0 | 0.0 | 0.0 | 0.0 | |
| 30 | LB+ | 0.0 | 0.0 | 0.0 | 0.0 | 0.0 | |
|  | LD | 0.0 | 0.0 | 0.0 | 0.0 | 0.0 | |
| 31 | LB+ | 0.0 | 0.0 | 0.0 | 0.0 | 0.0 | |
|  | LD | 0.0 | 0.0 | 0.0 | 0.0 | 0.0 | |
| 32 | LB+ | 0.0 | 0.0 | 0.0 | 0.0 | 0.0 | |
|  | LD | 0.0 | 0.0 | 0.0 | 0.0 | 0.0 | |
| 33 | LB+ | 0.0 | 0.0 | 0.0 | 0.0 | 0.0 | |
|  | LD | 0.0 | 0.0 | 0.0 | 0.0 | 0.0 | |
| 34 | LB+ | 0.0 | 0.0 | 0.0 | 0.0 | 0.0 | |
|  | LD | 0.0 | 0.0 | 0.0 | 0.0 | 0.0 | |
| 35 | LB+ | 0.0 | 0.0 | 0.0 | 0.0 | 0.0 | |
|  | LD | 0.0 | 0.0 | 0.0 | 0.0 | 0.0 | |
| 36 | LB+ | 0.0 | 0.0 | 0.0 | 0.0 | 0.0 | |
|  | LD | 0.0 | 0.0 | 0.0 | 0.0 | 0.0 | |
| 37 | LB+ | 0.0 | 0.5 | 0.0 | 0.0 | 0.5 | |
|  | LD | 0.0 | 0.0 | 0.0 | 0.0 | 0.0 | |
| 38 | LB+ | 0.0 | 0.5 | 0.0 | 0.0 | 0.5 | |
|  | LD | 0.0 | 0.0 | 0.0 | 0.0 | 0.0 | |
| 39 | LB+ | 0.0 | 4.0 | 0.5 | 0.0 | 4.0 | |
|  | LD | 0.0 | 0.0 | 0.0 | 0.0 | 0.0 | |
| 40 | LB+ | 0.0 | 1.5 | 0.0 | 0.0 | 1.5 | |
|  | LD | 0.0 | 0.0 | 0.0 | 0.0 | 0.0 | |
| 41 | LB+ | 0.0 | 2.0 | 0.0 | 0.0 | 2.0 | |
|  | LD | 0.0 | 0.0 | 0.0 | 0.0 | 0.0 | |
| 42 | LB+ | 0.0 | 3.5 | 2.0 | 0.0 | 5.0 | |
|  | LD | 0.0 | 0.0 | 0.0 | 0.0 | 0.0 | |
| 43 | LB+ | 0.0 | 6.5 | 0.0 | 0.0 | 6.5 | |
|  | LD | 0.0 | 0.0 | 0.0 | 0.0 | 0.0 | |
| 44 | LB+ | 0.0 | 11.0 | 1.5 | 0.0 | 12.0 | |
|  | LD | 0.0 | 0.0 | 0.0 | 0.0 | 0.0 | |
| 45 | LB+ | 0.0 | 11.0 | 3.5 | 0.0 | 12.0 | |
|  | LD | 0.0 | 0.0 | 0.0 | 0.0 | 0.0 | |
| 46 | LB+ | 0.0 | 11.5 | 3.5 | 0.0 | 12.0 | |
|  | LD | 0.0 | 0.0 | 0.0 | 0.0 | 0.0 | |
| 48 | LB+ | 0.0 | 22.0 | 9.0 | 0.0 | 24.5 | |
|  | LD | 0.0 | 0.0 | 0.0 | 0.0 | 0.0 | |
| 49 | LB+ | 0.0 | 41.5 | 9.0 | 0.0 | 41.5 | |
|  | LD | 0.0 | 0.0 | 0.0 | 0.0 | 0.0 | |
| 50 | LB+ | 0.0 | 51.0 | 26.0 | 0.0 | 53.5 | |
|  | LD | 0.0 | 0.0 | 0.0 | 0.0 | 0.0 | |
| 51 | LB+ | 0.0 | 64.5 | 35.0 | 0.0 | 65.0 | |
|  | LD | 0.0 | 0.0 | 0.0 | 0.0 | 0.0 | |
| 52 | LB+ | 0.0 | 62.0 | 49.0 | 0.0 | 65.0 | |
|  | LD | 0.0 | 0.0 | 0.0 | 0.0 | 0.0 | |
| 53 | LB+ | 0.0 | 67.0 | 54.0 | 0.0 | 67.5 | |
|  | LD | 0.0 | 0.0 | 0.0 | 0.0 | 0.0 | |
| 54 | LB+ | 0.0 | 74.0 | 60.5 | 0.0 | 74.5 | |
|  | LD | 0.0 | 0.0 | 0.0 | 0.0 | 0.0 | |
| 55 | LB+ | 0.0 | 71.5 | 59.0 | 0.0 | 72.0 | |
|  | LD | 0.0 | 0.0 | 0.0 | 0.0 | 0.0 | |
| 56 | LB+ | 0.5 | 74.5 | 67.5 | 0.5 | 75.5 | |
|  | LD | 0.0 | 0.0 | 0.0 | 0.0 | 0.0 | |
| 57 | LB+ | 0.5 | 81.0 | 72.5 | 0.0 | 81.5 | |
|  | LD | 0.0 | 0.0 | 0.0 | 0.0 | 0.0 | |
| 58 | LB+ | 0.0 | 78.5 | 75.5 | 0.0 | 80.0 | |
|  | LD | 0.0 | 0.0 | 0.0 | 0.0 | 0.0 | |
| 60 | LB+ | 0.0 | 83.0 | 77.5 | 0.0 | 84.0 |  |
|  | LD | 0.0 | 0.0 | 0.0 | 0.0 | 0.0 |  |
| 62 | LB+ | 0.0 | 79.5 | 81.0 | 1.0 | 81.5 |  |
|  | LD | 0.0 | 0.0 | 0.0 | 0.0 | 0.0 |  |
| 63 | LB+ | 0.0 | 80.5 | 82.5 | 0.5 | 82.5 |  |
|  | LD | 0.0 | 0.0 | 0.0 | 0.0 | 0.0 |  |
| 64 | LB+ | 0.0 | 80.5 | 87.5 | 0.5 | 87.0 |  |
|  | LD | 0.0 | 0.0 | 0.0 | 0.0 | 0.0 |  |
| 65 | LB+ | 0.0 | 83.0 | 88.0 | 0.0 | 89.0 |  |
|  | LD | 0.0 | 0.0 | 0.0 | 0.0 | 0.0 |  |
| 66 | LB+ | 0.0 | 80.5 | 82.5 | 0.0 | 83.0 |  |
|  | LD | 0.0 | 0.0 | 0.0 | 0.0 | 0.0 |  |
| 67 | LB+ | 0.5 | 89.0 | 90.0 | 0.0 | 90.0 |  |
|  | LD | 0.0 | 0.0 | 0.0 | 0.0 | 0.0 |  |
| 68 | LB+ | 0.0 | 83.0 | 88.0 | 0.5 | 89.0 |  |
|  | LD | 0.0 | 0.0 | 0.0 | 0.0 | 0.0 |  |
| 69 | LB+ | 0.0 | 85.5 | 87.5 | 1.0 | 88.0 |  |
|  | LD | 0.0 | 0.0 | 0.0 | 0.0 | 0.0 |  |

**Table S9.** Proportions of LB+ and LD hens (*n* = 200/hybrid strain) from batch 3 with severe plumage loss for four body regions. Min. one body region affected: proportion of hens with at least one body region affected by severe plumage loss.

| **Week of Life** | **Hybrid** | **Body Region** | | | | **Min. one body region affected** |
| --- | --- | --- | --- | --- | --- | --- |
|  |  | **Head/neck** | **Back** | **Tail** | **Wing** |  |
| 20 | LB+ | 0.0 | 0.0 | 0.0 | 0.0 | 0.0 |
|  | LD | 0.0 | 0.0 | 0.0 | 0.0 | 0.0 |
| 21 | LB+ | 0.0 | 0.0 | 0.0 | 0.0 | 0.0 |
|  | LD | 0.0 | 0.0 | 0.0 | 0.0 | 0.0 |
| 22 | LB+ | 0.0 | 0.0 | 0.0 | 0.0 | 0.0 |
|  | LD | 0.0 | 0.0 | 0.0 | 0.0 | 0.0 |
| 23 | LB+ | 0.0 | 0.0 | 0.0 | 0.0 | 0.0 |
|  | LD | 0.0 | 0.0 | 0.0 | 0.0 | 0.0 |
| 24 | LB+ | 0.0 | 0.0 | 0.0 | 0.0 | 0.0 |
|  | LD | 0.0 | 0.0 | 0.0 | 0.0 | 0.0 |
| 30 | LB+ | 0.0 | 0.0 | 0.0 | 0.0 | 0.0 |
|  | LD | 0.0 | 0.0 | 0.0 | 0.0 | 0.0 |
| 31 | LB+ | 0.0 | 0.0 | 0.0 | 0.0 | 0.0 |
|  | LD | 0.0 | 0.0 | 0.0 | 0.0 | 0.0 |
| 32 | LB+ | 0.0 | 0.0 | 0.0 | 0.0 | 0.0 |
|  | LD | 0.0 | 0.0 | 0.0 | 0.0 | 0.0 |
| 33 | LB+ | 0.0 | 0.0 | 0.0 | 0.0 | 0.0 |
|  | LD | 0.0 | 0.0 | 0.0 | 0.0 | 0.0 |
| 34 | LB+ | 0.0 | 0.0 | 0.0 | 0.0 | 0.0 |
|  | LD | 0.0 | 0.0 | 0.0 | 0.0 | 0.0 |
| 35 | LB+ | 0.0 | 0.0 | 0.0 | 0.0 | 0.0 |
|  | LD | 0.0 | 0.0 | 0.0 | 0.0 | 0.0 |
| 36 | LB+ | 0.0 | 0.0 | 0.0 | 0.0 | 0.0 |
|  | LD | 0.0 | 0.0 | 0.0 | 0.0 | 0.0 |
| 37 | LB+ | 0.0 | 0.0 | 0.0 | 0.0 | 0.0 |
|  | LD | 0.0 | 0.0 | 0.0 | 0.0 | 0.0 |
| 38 | LB+ | 0.0 | 1.0 | 0.0 | 0.0 | 1.0 |
|  | LD | 0.0 | 0.0 | 0.0 | 0.0 | 0.0 |
| 39 | LB+ | 0.0 | 3.0 | 0.0 | 0.0 | 3.0 |
|  | LD | 0.0 | 0.0 | 0.0 | 0.0 | 0.0 |
| 40 | LB+ | 0.0 | 2.0 | 0.5 | 0.0 | 2.5 |
|  | LD | 0.0 | 0.0 | 0.0 | 0.0 | 0.0 |
| 41 | LB+ | 0.0 | 1.0 | 0.5 | 0.0 | 1.5 |
|  | LD | 0.0 | 0.0 | 0.0 | 0.0 | 0.0 |
| 42 | LB+ | 0.0 | 4.5 | 3.0 | 0.0 | 6.0 |
|  | LD | 0.0 | 0.0 | 0.0 | 0.0 | 0.0 |
| 43 | LB+ | 0.0 | 7.0 | 2.5 | 0.0 | 7.0 |
|  | LD | 0.0 | 0.0 | 0.0 | 0.0 | 0.0 |
| 44 | LB+ | 0.0 | 10.0 | 2.5 | 0.0 | 11.5 |
|  | LD | 0.0 | 0.0 | 0.0 | 0.0 | 0.0 |
| 45 | LB+ | 0.0 | 15.0 | 5.0 | 0.0 | 16.0 |
|  | LD | 0.0 | 0.0 | 0.0 | 0.0 | 0.0 |
| 48 | LB+ | 0.0 | 25.0 | 32.5 | 0.0 | 40.0 |
|  | LD | 0.0 | 0.0 | 0.0 | 0.0 | 0.0 |
| 49 | LB+ | 0.5 | 35.0 | 43.0 | 0.0 | 49.5 |
|  | LD | 0.0 | 0.0 | 0.0 | 0.0 | 0.0 |
| 50 | LB+ | 0.0 | 56.5 | 58.5 | 0.0 | 68.0 |
|  | LD | 0.0 | 0.0 | 0.0 | 0.0 | 0.0 |
| 56 | LB+ | 0.0 | 62.5 | 68.5 | 0.0 | 73.5 |
|  | LD | 0.0 | 0.0 | 0.0 | 0.0 | 0.0 |

**Table S10.** Proportions of LB+ and LD hens (*n* = 200/hybrid strain) from batch 1 with injuries for five body regions. Min. one body region affected: proportion of hens with at least one body region affected by injuries.

| **Week of life** | **Hybrid** | **Body region** | | | | | **Min. one body region affected** |
| --- | --- | --- | --- | --- | --- | --- | --- |
|  |  | **Head/neck** | **Back** | **Tail** | **Wing** | **Breast/belly** |  |
| 20 | LB+ | 0.0 | 0.0 | 0.0 | 0.0 | 0.0 | 0.0 |
|  | LD | 0.0 | 0.0 | 0.0 | 0.0 | 0.0 | 0.0 |
| 21 | LB+ | 0.0 | 0.0 | 0.0 | 0.0 | 0.0 | 0.0 |
|  | LD | 0.0 | 0.0 | 0.0 | 0.0 | 0.0 | 0.0 |
| 22 | LB+ | 0.0 | 0.0 | 0.0 | 0.0 | 0.0 | 0.0 |
|  | LD | 0.0 | 0.0 | 0.0 | 0.0 | 0.0 | 0.0 |
| 23 | LB+ | 0.0 | 0.0 | 0.0 | 0.0 | 0.0 | 0.0 |
|  | LD | 0.0 | 0.0 | 0.0 | 0.0 | 0.0 | 0.0 |
| 24 | LB+ | 0.0 | 0.0 | 0.0 | 0.0 | 0.0 | 0.0 |
|  | LD | 0.0 | 0.0 | 0.0 | 0.0 | 0.0 | 0.0 |
| 25 | LB+ | 0.0 | 0.0 | 0.0 | 0.0 | 0.0 | 0.0 |
|  | LD | 0.0 | 0.0 | 0.0 | 0.0 | 0.0 | 0.0 |
| 26 | LB+ | 0.0 | 0.0 | 0.0 | 0.0 | 0.0 | 0.0 |
|  | LD | 0.0 | 0.0 | 0.0 | 0.0 | 0.0 | 0.0 |
| 27 | LB+ | 0.0 | 0.0 | 0.0 | 0.0 | 0.0 | 0.0 |
|  | LD | 0.0 | 0.0 | 0.0 | 0.0 | 0.0 | 0.0 |
| 28 | LB+ | 0.0 | 0.0 | 0.0 | 0.0 | 0.0 | 0.0 |
|  | LD | 0.0 | 0.0 | 0.0 | 0.0 | 0.0 | 0.0 |
| 29 | LB+ | 0.0 | 0.0 | 0.0 | 0.0 | 0.0 | 0.0 |
|  | LD | 0.0 | 0.0 | 0.0 | 0.0 | 0.0 | 0.0 |
| 30 | LB+ | 0.0 | 0.0 | 0.0 | 0.0 | 0.0 | 0.0 |
|  | LD | 0.0 | 0.0 | 0.0 | 0.0 | 0.0 | 0.0 |
| 31 | LB+ | 0.0 | 0.0 | 0.0 | 0.0 | 0.0 | 0.0 |
|  | LD | 0.0 | 0.0 | 0.0 | 0.0 | 0.0 | 0.0 |
| 32 | LB+ | 0.0 | 0.0 | 0.0 | 0.0 | 0.0 | 0.0 |
|  | LD | 0.0 | 0.0 | 0.0 | 0.0 | 0.0 | 0.0 |
| 33 | LB+ | 0.0 | 0.0 | 0.0 | 0.0 | 0.0 | 0.0 |
|  | LD | 0.0 | 0.0 | 0.0 | 0.0 | 0.0 | 0.0 |
| 34 | LB+ | 0.0 | 0.0 | 0.0 | 0.0 | 0.0 | 0.0 |
|  | LD | 0.0 | 0.0 | 0.0 | 0.0 | 0.0 | 0.0 |
| 35 | LB+ | 0.0 | 0.0 | 0.0 | 0.0 | 0.0 | 0.0 |
|  | LD | 0.0 | 0.0 | 0.0 | 0.0 | 0.0 | 0.0 |
| 36 | LB+ | 0.0 | 0.0 | 0.0 | 0.0 | 0.0 | 0.0 |
|  | LD | 0.0 | 0.0 | 0.0 | 0.0 | 0.0 | 0.0 |
| 37 | LB+ | 0.0 | 0.0 | 0.0 | 0.0 | 0.0 | 0.0 |
|  | LD | 0.0 | 0.0 | 0.0 | 0.0 | 0.0 | 0.0 |
| 38 | LB+ | 0.0 | 0.0 | 0.0 | 0.0 | 0.0 | 0.0 |
|  | LD | 0.0 | 0.0 | 0.0 | 0.0 | 0.0 | 0.0 |
| 39 | LB+ | 0.0 | 0.0 | 0.0 | 0.0 | 0.0 | 0.0 |
|  | LD | 0.0 | 0.0 | 0.0 | 0.0 | 0.0 | 0.0 |
| 40 | LB+ | 0.0 | 0.0 | 0.0 | 0.0 | 0.0 | 0.0 |
|  | LD | 0.0 | 0.0 | 0.0 | 0.0 | 0.0 | 0.0 |
| 41 | LB+ | 0.0 | 0.5 | 0.0 | 0.0 | 0.0 | 0.5 |
|  | LD | 0.0 | 0.0 | 0.0 | 0.0 | 0.0 | 0.0 |
| 42 | LB+ | 0.0 | 0.0 | 0.0 | 0.5 | 0.0 | 0.5 |
|  | LD | 0.0 | 0.0 | 0.0 | 0.0 | 0.0 | 0.0 |
| 43 | LB+ | 0.0 | 0.0 | 0.0 | 0.0 | 0.0 | 0.0 |
|  | LD | 0.0 | 0.0 | 0.0 | 0.0 | 0.0 | 0.0 |
| 44 | LB+ | 0.0 | 0.0 | 0.0 | 0.0 | 0.0 | 0.0 |
|  | LD | 0.0 | 0.0 | 0.0 | 0.0 | 0.0 | 0.0 |
| 45 | LB+ | 0.0 | 0.0 | 0.0 | 0.0 | 0.0 | 0.0 |
|  | LD | 0.0 | 0.0 | 0.0 | 0.0 | 0.0 | 0.0 |
| 46 | LB+ | 1.0 | 0.0 | 0.0 | 0.0 | 0.0 | 1.0 |
|  | LD | 0.0 | 0.0 | 0.0 | 0.0 | 0.0 | 0.0 |
| 47 | LB+ | 0.0 | 0.5 | 0.0 | 0.0 | 0.0 | 0.5 |
|  | LD | 0.0 | 0.0 | 0.0 | 0.0 | 0.0 | 0.0 |
| 48 | LB+ | 0.0 | 0.0 | 0.0 | 0.0 | 0.0 | 0.0 |
|  | LD | 0.0 | 0.0 | 0.0 | 0.0 | 0.0 | 0.0 |
| 49 | LB+ | 0.0 | 1.0 | 0.0 | 0.0 | 0.0 | 1.0 |
|  | LD | 0.0 | 0.0 | 0.0 | 0.0 | 0.0 | 0.0 |
| 50 | LB+ | 0.0 | 0.5 | 0.0 | 0.5 | 0.0 | 1.0 |
|  | LD | 0.0 | 0.0 | 0.0 | 0.0 | 0.0 | 0.0 |
| 51 | LB+ | 0.0 | 2.0 | 0.0 | 0.0 | 0.0 | 2.0 |
|  | LD | 0.0 | 0.0 | 0.0 | 0.0 | 0.0 | 0.0 |
| 52 | LB+ | 0.0 | 1.5 | 0.0 | 0.0 | 0.0 | 1.5 |
|  | LD | 0.0 | 0.0 | 0.0 | 0.0 | 0.0 | 0.0 |
| 53 | LB+ | 0.0 | 2.0 | 0.0 | 0.0 | 0.0 | 2.0 |
|  | LD | 0.0 | 0.0 | 0.0 | 0.0 | 0.0 | 0.0 |
| 54 | LB+ | 0.0 | 1.5 | 0.0 | 0.0 | 0.0 | 1.5 |
|  | LD | 0.0 | 0.0 | 0.0 | 0.0 | 0.0 | 0.0 |
| 55 | LB+ | 0.0 | 1.5 | 0.0 | 0.0 | 0.0 | 1.5 |
|  | LD | 0.0 | 0.0 | 0.0 | 0.0 | 0.0 | 0.0 |
| 56 | LB+ | 0.0 | 0.5 | 0.0 | 0.0 | 0.0 | 0.5 |
|  | LD | 0.0 | 0.0 | 0.0 | 0.0 | 0.0 | 0.0 |
| 57 | LB+ | 0.0 | 1.5 | 0.5 | 0.5 | 0.0 | 2.5 |
|  | LD | 0.0 | 0.0 | 0.0 | 0.0 | 0.0 | 0.0 |
| 58 | LB+ | 0.0 | 2.5 | 0.0 | 0.0 | 0.0 | 2.5 |
|  | LD | 0.0 | 0.0 | 0.0 | 0.0 | 0.0 | 0.0 |
| 59 | LB+ | 0.0 | 1.5 | 1.0 | 0.0 | 0.0 | 2.5 |
|  | LD | 0.0 | 0.0 | 0.0 | 0.0 | 0.0 | 0.0 |
| 60 | LB+ | 0.0 | 2.0 | 1.5 | 0.0 | 0.0 | 3.5 |
|  | LD | 0.0 | 0.0 | 0.0 | 0.0 | 0.0 | 0.0 |
| 61 | LB+ | 1.0 | 2.5 | 1.5 | 0.0 | 1.0 | 6.0 |
|  | LD | 0.0 | 0.0 | 0.0 | 0.0 | 0.0 | 0.0 |
| 62 | LB+ | 0.0 | 1.0 | 2.0 | 0.0 | 0.0 | 3.0 |
|  | LD | 0.0 | 0.0 | 0.0 | 0.0 | 0.0 | 0.0 |
| 63 | LB+ | 0.0 | 1.0 | 1.5 | 0.0 | 7.5 | 10.0 |
|  | LD | 0.0 | 0.0 | 0.0 | 0.0 | 0.0 | 0.0 |
| 64 | LB+ | 0.0 | 1.0 | 5.0 | 0.5 | 3.5 | 9.0 |
|  | LD | 0.0 | 0.0 | 0.0 | 0.0 | 0.0 | 0.0 |
| 65 | LB+ | 0.0 | 1.5 | 0.5 | 0.0 | 4.0 | 5.5 |
|  | LD | 0.0 | 0.0 | 0.0 | 0.0 | 0.0 | 0.0 |
| 66 | LB+ | 0.0 | 1.0 | 1.0 | 0.0 | 4.5 | 6.0 |
|  | LD | 0.0 | 0.0 | 0.0 | 0.0 | 0.0 | 0.0 |
| 67 | LB+ | 0.0 | 0.0 | 1.0 | 0.0 | 1.0 | 2.0 |
|  | LD | 0.0 | 0.0 | 0.0 | 0.0 | 0.0 | 0.0 |
| 68 | LB+ | 0.0 | 0.0 | 0.5 | 0.0 | 3.0 | 3.5 |
|  | LD | 0.0 | 0.0 | 0.0 | 0.0 | 0.0 | 0.0 |
| 69 | LB+ | 0.0 | 1.5 | 0.0 | 0.0 | 4.0 | 5.5 |
|  | LD | 0.0 | 0.0 | 0.0 | 0.0 | 0.0 | 0.0 |
| 70 | LB+ | 0.0 | 0.0 | 1.5 | 0.0 | 1.5 | 3.0 |
|  | LD | 0.0 | 0.0 | 0.0 | 0.0 | 0.0 | 0.0 |
| 71 | LB+ | 0.0 | 0.0 | 0.5 | 0.0 | 2.0 | 2.5 |
|  | LD | 0.0 | 0.0 | 0.0 | 0.0 | 0.0 | 0.0 |

**Table S11.** Proportions of LB+ and LD hens (*n* = 200/hybrid strain) from batch 2 with injuries for five body regions. Min. one body region affected: proportion of hens with at least one body region affected by injuries.

| **Week of life** | **Hybrid** | **Body region** | | | | | **Min. one body region affected** |
| --- | --- | --- | --- | --- | --- | --- | --- |
|  |  | **Head/neck** | **Back** | **Tail** | **Wing** | **Breast/belly** |  |
| 20 | LB+ | 0.0 | 0.0 | 0.0 | 0.0 | 0.0 | 0.0 |
|  | LD | 0.0 | 0.0 | 0.0 | 0.0 | 0.0 | 0.0 |
| 21 | LB+ | 0.0 | 0.0 | 0.0 | 0.0 | 0.0 | 0.0 |
|  | LD | 0.0 | 0.0 | 0.0 | 0.0 | 0.0 | 0.0 |
| 22 | LB+ | 0.0 | 0.0 | 0.0 | 0.0 | 0.0 | 0.0 |
|  | LD | 0.0 | 0.0 | 0.0 | 0.0 | 0.0 | 0.0 |
| 23 | LB+ | 0.0 | 0.0 | 0.0 | 0.0 | 0.0 | 0.0 |
|  | LD | 0.0 | 0.0 | 0.0 | 0.0 | 0.0 | 0.0 |
| 24 | LB+ | 0.0 | 0.0 | 0.0 | 0.0 | 0.0 | 0.0 |
|  | LD | 0.0 | 0.0 | 0.0 | 0.0 | 0.0 | 0.0 |
| 25 | LB+ | 0.0 | 0.0 | 0.0 | 0.0 | 0.0 | 0.0 |
|  | LD | 0.0 | 0.0 | 0.0 | 0.0 | 0.0 | 0.0 |
| 26 | LB+ | 0.0 | 0.0 | 0.0 | 0.0 | 0.0 | 0.0 |
|  | LD | 0.0 | 0.0 | 0.0 | 0.0 | 0.0 | 0.0 |
| 27 | LB+ | 0.0 | 0.0 | 0.0 | 0.0 | 0.0 | 0.0 |
|  | LD | 0.0 | 0.0 | 0.0 | 0.0 | 0.0 | 0.0 |
| 28 | LB+ | 0.0 | 0.0 | 0.0 | 0.0 | 0.0 | 0.0 |
|  | LD | 0.0 | 0.0 | 0.0 | 0.0 | 0.0 | 0.0 |
| 29 | LB+ | 0.0 | 0.0 | 0.0 | 0.0 | 0.0 | 0.0 |
|  | LD | 0.0 | 0.0 | 0.0 | 0.0 | 0.0 | 0.0 |
| 30 | LB+ | 0.0 | 0.0 | 0.0 | 0.0 | 0.0 | 0.0 |
|  | LD | 0.0 | 0.0 | 0.0 | 0.0 | 0.0 | 0.0 |
| 31 | LB+ | 0.0 | 0.0 | 0.0 | 0.0 | 0.0 | 0.0 |
|  | LD | 0.0 | 0.0 | 0.0 | 0.0 | 0.0 | 0.0 |
| 32 | LB+ | 0.0 | 0.0 | 0.0 | 0.0 | 0.0 | 0.0 |
|  | LD | 0.0 | 0.0 | 0.0 | 0.0 | 0.0 | 0.0 |
| 33 | LB+ | 0.0 | 0.0 | 0.0 | 0.0 | 0.0 | 0.0 |
|  | LD | 0.0 | 0.0 | 0.0 | 0.0 | 0.0 | 0.0 |
| 34 | LB+ | 0.0 | 0.0 | 0.0 | 0.0 | 0.0 | 0.0 |
|  | LD | 0.0 | 0.0 | 0.0 | 0.0 | 0.0 | 0.0 |
| 35 | LB+ | 0.0 | 0.0 | 0.0 | 0.0 | 0.0 | 0.0 |
|  | LD | 0.0 | 0.0 | 0.0 | 0.0 | 0.0 | 0.0 |
| 36 | LB+ | 0.0 | 0.0 | 0.0 | 0.0 | 0.0 | 0.0 |
|  | LD | 0.0 | 0.0 | 0.0 | 0.0 | 0.0 | 0.0 |
| 37 | LB+ | 0.0 | 0.0 | 0.0 | 0.0 | 0.0 | 0.0 |
|  | LD | 0.0 | 0.0 | 0.0 | 0.0 | 0.0 | 0.0 |
| 38 | LB+ | 0.0 | 1.0 | 1.0 | 0.0 | 0.0 | 2.0 |
|  | LD | 0.0 | 0.0 | 0.0 | 0.0 | 0.0 | 0.0 |
| 39 | LB+ | 0.0 | 0.0 | 2.0 | 0.0 | 0.0 | 2.0 |
|  | LD | 0.0 | 0.0 | 0.0 | 0.0 | 0.0 | 0.0 |
| 40 | LB+ | 0.0 | 2.0 | 1.0 | 0.0 | 1.0 | 4.0 |
|  | LD | 0.0 | 0.0 | 0.0 | 0.0 | 0.0 | 0.0 |
| 41 | LB+ | 0.0 | 0.0 | 0.0 | 0.0 | 3.0 | 3.0 |
|  | LD | 0.0 | 0.0 | 0.0 | 0.0 | 0.0 | 0.0 |
| 42 | LB+ | 0.0 | 2.0 | 0.0 | 0.0 | 3.0 | 4.5 |
|  | LD | 0.0 | 0.0 | 0.0 | 0.0 | 0.0 | 0.0 |
| 43 | LB+ | 0.0 | 1.5 | 1.0 | 0.0 | 2.5 | 5.0 |
|  | LD | 0.0 | 0.0 | 0.0 | 0.0 | 0.0 | 0.0 |
| 44 | LB+ | 0.0 | 0.5 | 0.5 | 0.0 | 5.0 | 6.0 |
|  | LD | 0.0 | 0.0 | 0.0 | 0.0 | 0.0 | 0.0 |
| 45 | LB+ | 0.0 | 0.5 | 0.5 | 0.0 | 3.5 | 3.5 |
|  | LD | 0.0 | 0.0 | 0.0 | 0.0 | 0.0 | 0.0 |
| 46 | LB+ | 0.0 | 0.0 | 0.0 | 0.0 | 6.0 | 6.0 |
|  | LD | 0.0 | 0.0 | 0.0 | 0.0 | 0.0 | 0.0 |
| 48 | LB+ | 0.0 | 0.0 | 1.0 | 0.0 | 10.0 | 11.0 |
|  | LD | 0.0 | 0.0 | 0.0 | 0.0 | 0.0 | 0.0 |
| 49 | LB+ | 0.0 | 0.0 | 0.0 | 0.0 | 5.5 | 5.5 |
|  | LD | 0.0 | 0.0 | 0.0 | 0.0 | 0.0 | 0.0 |
| 50 | LB+ | 0.0 | 0.0 | 0.0 | 0.0 | 6.0 | 6.0 |
|  | LD | 0.0 | 0.0 | 0.0 | 0.0 | 0.0 | 0.0 |
| 51 | LB+ | 0.0 | 0.0 | 0.0 | 0.0 | 7.0 | 7.0 |
|  | LD | 0.0 | 0.0 | 0.0 | 0.0 | 0.0 | 0.0 |
| 52 | LB+ | 0.0 | 0.0 | 0.0 | 0.0 | 8.0 | 8.0 |
|  | LD | 0.0 | 0.0 | 0.0 | 0.0 | 0.0 | 0.0 |
| 53 | LB+ | 0.0 | 0.0 | 0.0 | 0.0 | 8.0 | 8.0 |
|  | LD | 0.0 | 0.0 | 0.0 | 0.0 | 0.0 | 0.0 |
| 54 | LB+ | 0.0 | 0.0 | 1.0 | 0.0 | 7.5 | 8.0 |
|  | LD | 0.0 | 0.0 | 0.0 | 0.0 | 0.0 | 0.0 |
| 55 | LB+ | 0.0 | 0.0 | 0.0 | 0.0 | 9.0 | 9.0 |
|  | LD | 0.0 | 0.0 | 0.0 | 0.0 | 0.0 | 0.0 |
| 56 | LB+ | 0.0 | 1.0 | 0.5 | 0.0 | 9.5 | 11.0 |
|  | LD | 0.0 | 0.0 | 0.0 | 0.0 | 0.0 | 0.0 |
| 57 | LB+ | 0.0 | 0.0 | 1.0 | 0.0 | 9.5 | 10.0 |
|  | LD | 0.0 | 0.0 | 0.0 | 0.0 | 0.0 | 0.0 |
| 58 | LB+ | 0.0 | 0.0 | 0.0 | 0.5 | 11.5 | 11.5 |
|  | LD | 0.0 | 0.0 | 0.0 | 0.0 | 0.0 | 0.0 |
| 60 | LB+ | 0.0 | 0.5 | 0.5 | 0.0 | 11.0 | 11.5 |
|  | LD | 0.0 | 0.0 | 0.0 | 0.0 | 0.0 | 0.0 |
| 62 | LB+ | 0.0 | 0.0 | 0.5 | 0.0 | 9.5 | 10.0 |
|  | LD | 0.0 | 0.0 | 0.0 | 0.0 | 0.0 | 0.0 |
| 63 | LB+ | 0.0 | 0.0 | 2.0 | 0.0 | 7.0 | 9.0 |
|  | LD | 0.0 | 0.0 | 0.0 | 0.0 | 0.0 | 0.0 |
| 64 | LB+ | 0.0 | 0.0 | 1.5 | 0.0 | 5.0 | 6.5 |
|  | LD | 0.0 | 0.0 | 0.0 | 0.0 | 0.0 | 0.0 |
| 65 | LB+ | 0.0 | 1.0 | 0.5 | 0.0 | 8.0 | 9.0 |
|  | LD | 0.0 | 0.0 | 0.0 | 0.0 | 0.0 | 0.0 |
| 66 | LB+ | 0.0 | 0.0 | 1.0 | 0.0 | 7.0 | 8.0 |
|  | LD | 0.0 | 0.0 | 0.0 | 0.0 | 0.0 | 0.0 |
| 67 | LB+ | 0.0 | 0.0 | 0.0 | 0.0 | 7.5 | 7.5 |
|  | LD | 0.0 | 0.0 | 0.0 | 0.0 | 0.0 | 0.0 |
| 68 | LB+ | 0.0 | 0.0 | 0.5 | 0.0 | 8.0 | 8.5 |
|  | LD | 0.0 | 0.0 | 0.0 | 0.0 | 0.0 | 0.0 |
| 69 | LB+ | 0.0 | 1.0 | 0.0 | 0.0 | 8.0 | 8.5 |
|  | LD | 0.0 | 0.0 | 0.0 | 0.0 | 0.0 | 0.0 |

**Table S12.** Proportions of LB+ and LD hens (*n* = 200/hybrid strain) from batch 3 with injuries for five body regions. Min. one body region affected: proportion of hens with at least one body region affected by injuries.

| **Week of life** | **Hybrid** | **Body region** | | | | | **Min. one body region affected** |
| --- | --- | --- | --- | --- | --- | --- | --- |
|  |  | **Head/neck** | **Back** | **Tail** | **Wing** | **Breast/belly** |  |
| 20 | LB+ | 0.0 | 0.0 | 0.0 | 0.0 | 0.0 | 0.0 |
|  | LD | 0.0 | 0.0 | 0.0 | 0.0 | 0.0 | 0.0 |
| 21 | LB+ | 0.0 | 0.0 | 0.0 | 0.0 | 0.0 | 0.0 |
|  | LD | 0.0 | 0.0 | 0.0 | 0.0 | 0.0 | 0.0 |
| 22 | LB+ | 0.0 | 0.0 | 0.0 | 0.0 | 0.0 | 0.0 |
|  | LD | 0.0 | 0.0 | 0.0 | 0.0 | 0.0 | 0.0 |
| 23 | LB+ | 0.0 | 0.0 | 0.0 | 0.0 | 0.0 | 0.0 |
|  | LD | 0.0 | 0.0 | 0.0 | 0.0 | 0.0 | 0.0 |
| 24 | LB+ | 0.0 | 0.0 | 0.0 | 0.0 | 0.0 | 0.0 |
|  | LD | 0.0 | 0.0 | 0.0 | 0.0 | 0.0 | 0.0 |
| 30 | LB+ | 0.0 | 0.0 | 0.0 | 0.0 | 0.0 | 0.0 |
|  | LD | 0.0 | 0.0 | 0.0 | 0.0 | 0.0 | 0.0 |
| 31 | LB+ | 0.0 | 0.0 | 0.0 | 0.0 | 0.0 | 0.0 |
|  | LD | 0.0 | 0.0 | 0.0 | 0.0 | 0.0 | 0.0 |
| 32 | LB+ | 0.0 | 0.0 | 0.0 | 0.0 | 0.0 | 0.0 |
|  | LD | 0.0 | 0.0 | 0.0 | 0.0 | 0.0 | 0.0 |
| 33 | LB+ | 0.0 | 0.0 | 0.0 | 0.0 | 0.0 | 0.0 |
|  | LD | 0.0 | 0.0 | 0.0 | 0.0 | 0.0 | 0.0 |
| 34 | LB+ | 0.0 | 0.0 | 0.0 | 0.0 | 0.0 | 0.0 |
|  | LD | 0.0 | 0.0 | 0.0 | 0.0 | 0.0 | 0.0 |
| 35 | LB+ | 0.0 | 0.0 | 0.0 | 0.0 | 0.0 | 0.0 |
|  | LD | 0.0 | 0.0 | 0.0 | 0.0 | 0.0 | 0.0 |
| 36 | LB+ | 0.0 | 0.0 | 0.5 | 0.0 | 1.5 | 2.0 |
|  | LD | 0.0 | 0.0 | 0.0 | 0.0 | 0.0 | 0.0 |
| 37 | LB+ | 0.0 | 0.0 | 0.0 | 0.0 | 2.5 | 2.5 |
|  | LD | 0.0 | 0.0 | 0.0 | 0.0 | 0.0 | 0.0 |
| 38 | LB+ | 0.0 | 0.0 | 0.0 | 0.0 | 1.0 | 1.0 |
|  | LD | 0.0 | 0.0 | 0.0 | 0.0 | 0.0 | 0.0 |
| 39 | LB+ | 0.0 | 0.0 | 0.0 | 0.0 | 2.5 | 2.5 |
|  | LD | 0.0 | 0.0 | 0.0 | 0.0 | 0.0 | 0.0 |
| 40 | LB+ | 0.0 | 0.0 | 0.0 | 0.0 | 3.0 | 3.0 |
|  | LD | 0.0 | 0.0 | 0.0 | 0.0 | 0.0 | 0.0 |
| 41 | LB+ | 0.0 | 0.0 | 0.0 | 0.0 | 3.5 | 3.5 |
|  | LD | 0.0 | 0.0 | 0.0 | 0.0 | 0.0 | 0.0 |
| 42 | LB+ | 0.0 | 0.0 | 0.0 | 0.0 | 1.0 | 1.0 |
|  | LD | 0.0 | 0.0 | 0.0 | 0.0 | 0.0 | 0.0 |
| 43 | LB+ | 0.0 | 0.0 | 0.0 | 0.0 | 3.0 | 3.0 |
|  | LD | 0.0 | 0.0 | 0.0 | 0.0 | 0.5 | 0.5 |
| 44 | LB+ | 0.0 | 0.0 | 0.0 | 0.0 | 4.0 | 4.0 |
|  | LD | 0.0 | 0.0 | 0.0 | 0.0 | 0.0 | 0.0 |
| 45 | LB+ | 0.5 | 0.0 | 0.5 | 0.0 | 2.5 | 3.5 |
|  | LD | 0.0 | 0.0 | 0.0 | 0.0 | 0.0 | 0.0 |
| 48 | LB+ | 0.0 | 0.5 | 0.5 | 0.0 | 5.0 | 5.5 |
|  | LD | 0.0 | 0.0 | 0.0 | 0.0 | 0.0 | 0.0 |
| 49 | LB+ | 0.0 | 0.0 | 0.5 | 0.0 | 7.5 | 8.0 |
|  | LD | 0.0 | 0.0 | 0.0 | 0.0 | 0.0 | 0.0 |
| 50 | LB+ | 0.0 | 0.5 | 1.0 | 0.0 | 6.0 | 7.0 |
|  | LD | 0.0 | 0.0 | 0.0 | 0.0 | 0.0 | 0.0 |
| 56 | LB+ | 0.0 | 0.0 | 0.5 | 0.0 | 7.5 | 8.0 |
|  | LD | 0.0 | 0.0 | 0.0 | 0.0 | 0.0 | 0.0 |
